# SARS‐CoV‐2 enhances lysosomal exocytosis and deacidifies lysosomes to facilitate viral release

**DOI:** 10.1002/mlf2.70065

**Published:** 2026-06-26

**Authors:** Fujun Qin, Chuang Yan, Zizheng Liu, Dianbing Wang, Huimin Zhong, Qiang Ding, Minghai Chen, Xian‐En Zhang

**Affiliations:** ^1^ State Key Laboratory of Quantitative Synthetic Biology, Shenzhen Institute of Synthetic Biology, Shenzhen Institutes of Advanced Technology Chinese Academy of Sciences Shenzhen China; ^2^ Faculty of Synthetic Biology Shenzhen University of Advanced Technology Shenzhen China; ^3^ State Key Laboratory of Biomacromolecules, Institute of Biophysics Chinese Academy of Sciences Beijing China; ^4^ School of Medicine Tsinghua University Beijing China

**Keywords:** lysosomal exocytosis, lysosome deacidification, ORF3a protein, SARS‐CoV‐2, synthetic biology

## Abstract

The mechanism of SARS‐CoV‐2 egress predominantly governs the quantity and quality of progeny viruses, thereby significantly contributing to viral pathogenicity. However, the key factors influencing viral egress remain largely unclear. In this study, using transcription‐ and replication‐competent SARS‐CoV‐2 virus‐like‐particle (SARS‐CoV‐2 trVLP), electron microscopy, drug inhibition assays, and cellular pH‐sensitive fluorescent probes, we demonstrate that increased lysosomal exocytosis efficiency and lysosome deacidification play a pivotal role in facilitating SARS‐CoV‐2 egress. Specifically, SARS‐CoV‐2 may use multiple egress pathways, with lysosomal exocytosis as the primary mechanism and the biosynthetic secretory pathway as a less efficient route. Viral infection enhances lysosomal exocytosis via the ORF3a protein, thus facilitating viral release. SARS‐CoV‐2 infection also induces lysosome deacidification; moreover, treatment with bafilomycin A1, which induces lysosome deacidification, further enhances viral egress. Furthermore, we systematically investigate how viral proteins affect lysosomal pH and enzymatic activities. Our findings reveal that ORF3a and E proteins induce lysosome deacidification and diminish lysosomal enzyme activities, probably protecting progeny viruses from premature cleavage and degradation. This study provides mechanistic insight into how SARS‐CoV‐2 promotes lysosomal exocytosis and triggers lysosome deacidification for viral release.

## Impact statement

The development of effective drugs against SARS‐CoV‐2 has been hindered by an incomplete understanding of the virus–host interaction mechanisms. Here, we demonstrate that the efficiency of lysosomal exocytosis and lysosomal pH critically influence both the release and infectivity of SARS‐CoV‐2 progeny viruses. Specifically, SARS‐CoV‐2 promotes lysosomal exocytosis via ORF3a to facilitate viral egress. Lysosomes are deacidified and lysosomal enzymatic function is impaired by the ORF3a and E proteins, which may help prevent newly formed virions from undergoing premature proteolytic degradation. Targeting the ORF3a‐promoted lysosomal exocytosis pathway may represent a promising approach to inhibit SARS‐CoV‐2 egress and facilitate the development of novel antiviral therapies.

## INTRODUCTION

The COVID‐19 pandemic has significantly impacted global health, with sporadic infections now representing a prevalent trend^1^, ^2^, ^3^. In‐depth investigations have elucidated the mechanisms of SARS‐CoV‐2 cell entry, genomic replication, and assembly^4^, ^5^. However, the specific egress pathway of SARS‐CoV‐2 and the determining factors influencing viral egress remain elusive. There are two primary pathways for coronavirus egress. One is the biosynthetic secretory pathway, which delivers cargo from the endoplasmic reticulum (ER) via the Golgi and secretory vesicles to the extracellular space or specific intracellular locations. The biosynthetic secretory pathway is used by most the enveloped viruses^6^, ^7^, ^8^, such as SARS‐CoV and infectious bronchitis virus^9^, ^10^. The alternative mechanism involves lysosomal exocytosis, a pathway typically utilized by a minority of viruses^11^, including but not limited to mouse hepatitis virus (MHV) and porcine hemagglutinating encephalomyelitis virus (PHEV)^12^, ^13^. Early studies showed that SARS‐CoV‐2 virus particles were transported into lysosome‐like vesicles and lysosomal‐associated membrane protein 1 (LAMP1)‐positive organelles, suggesting that SARS‐CoV‐2 progeny virus particles were released by lysosomal exocytosis^12^, ^14^. However, subsequent studies showed that SARS‐CoV‐2 exited the cell through single‐membrane vesicles and distal secretory organelles, implying that SARS‐CoV‐2 was released through the secretory pathway^15^, ^16^. In addition, since SARS‐CoV‐2 progeny viruses are released as early as 6 hours post infection (hpi), ultrastructural studies conducted after this time point may include both released progeny viruses (egress) and progeny viruses that are subsequently internalized by infected or neighboring cells (uptake)^14^, making it difficult to delineate these pathways. This divergence in findings contributes to the ongoing debate about the precise mechanisms of SARS‐CoV‐2 egress.

Viruses utilizing lysosomal exocytosis for egress can alter lysosomal pH by either deacidifying or acidifying lysosomes, with each alteration potentially causing distinct disruptions in lysosomal function. For example, MHV infection leads to lysosome deacidification, thereby inactivating lysosomal proteases and disrupting antigen processing and presentation^12^. In contrast, PHEV infection leads to lysosome acidification and the activation of lysosomal degradation enzymes. Inhibition of lysosomal exocytosis hinders PHEV infection, and lysosomal acidification is essential for PHEV egress. Despite the opposite lysosomal pH and activity triggered by MHV and PHEV, they both induce lysosomal dysfunction in ways that are currently undefined.

To determine the exact mechanism of SARS‐CoV‐2 egress and identify the key factors influencing viral egress, we used a trans‐complementation system for the production of transcription‐ and replication‐competent SARS‐CoV‐2 virus‐like‐particles (SARS‐CoV‐2 trVLP)^17^. In this system, the viral nucleocapsid (N) gene was replaced with a green fluorescent protein (GFP) reporter gene; consequently, the SARS‐CoV‐2 trVLP cycle can only be completed in host cells expressing the N protein (Caco‐2‐N)^18^. Hence, the SARS‐CoV‐2 trVLP system could serve as an ideal pseudoviral model for studying viral replication and egress. In this study, using the SARS‐CoV‐2 trVLP and drug inhibition assays, we demonstrated that SARS‐CoV‐2 trVLP utilized lysosomal exocytosis for egress, a process enhanced by the ORF3a protein, and lysosome deacidification facilitated viral egress. Furthermore, using pH‐sensitive fluorescent probes and molecular imaging techniques, we found that SARS‐CoV‐2 ORF3a and E proteins triggered lysosome deacidification and attenuated the activities of lysosomal enzymes, thereby potentially protecting progeny viruses from premature cleavage and degradation.

## RESULTS

### Ultrastructural characterization of SARS‐CoV‐2 egress based on exocytosis inhibitors

**Figure 1:**
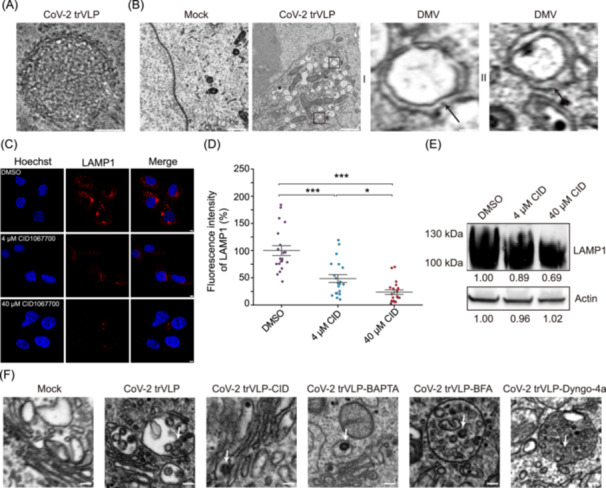
Ultrastructural characterization of SARS‐CoV‐2 egress based on exocytosis inhibitors. (A) Transmission electron microscopy (TEM) of purified SARS‐CoV‐2 trVLP. Representative image illustrates viral morphology. The images were acquired at a magnification of 110,000×. Scale bar, 50 nm. (B) Scanning electron microscopy (SEM) of SARS‐CoV‐2 trVLP‐infected cells. SEM images demonstrate a significant presence of double‐membrane vesicles (DMVs) in SARS‐CoV‐2 trVLP‐infected cells compared to mock‐infected controls. Black arrows highlight DMVs. The images were acquired at a magnification of 32,000×. Scale bars, 1 μm (left and middle images) and 100 nm (right images I and II). (C) Impact of lysosomal exocytosis inhibitor CID1067700 on the formation of lysosomes in Caco‐2‐N cells by immunofluorescence. Representative confocal microscopy images of Caco‐2‐N cells treated with DMSO or CID1067700 (4 or 40 µM) for 6 h and immunostained with the LAMP1 antibody are shown. Scale bar, 5 µm. (D) Quantitative analysis of the effect of CID1067700 on lysosome formation in Caco‐2‐N cells, performed by measuring the fluorescence intensity of LAMP1. Cells were treated with DMSO (as the solvent control) or CID1067700 (*n* = 20 cells/group). Cells were selected from random microscopic fields, and analyses were performed under blinded conditions. LAMP1 fluorescence intensity in DMSO‐treated cells was normalized to 100%. (E) Effects of CID1067700 on intracellular LAMP1 in SARS‐CoV‐2 trVLP‐infected cells (8–14 hpi) detected by Western blot. DMSO treatment served as a negative control. (F) SEM of SARS‐CoV‐2 trVLP‐infected cells. Representative SEM images depict SARS‐CoV‐2 trVLP‐infected cells treated with various drugs. Dyngo‐4a (30 µM) was applied from 6 to 14 hpi. Brefeldin A (BFA, 5 µg/ml), CID1067700 (40 µM), and BAPTA‐AM (30 µM) were added from 8 to 14 hpi. White arrows indicate SARS‐CoV‐2 trVLP particles. The images were acquired at a magnification of 32,000×. Scale bar, 100 nm. Significance was assessed using one‐way ANOVA (D). *p < 0.05; ***p < 0.001. ANOVA, analysis of variance; DMSO, dimethyl sulfoxide; hpi, hours post infection.

Since the mechanism of SARS‐CoV‐2 release from infected cells was still under debate^12^, ^14^, ^15^, ^16^, we investigated the egress pathway of SARS‐CoV‐2 at the ultrastructural level based on multiple exocytosis inhibitors. The SARS‐CoV‐2 trVLP used in this study showed a viral morphology indistinguishable from the authentic virus (Figure 1A). Moreover, SARS‐CoV‐2 trVLP‐infected cells produced double‐membrane vesicles (DMVs) (Figure 1B), which were the primary replication organelles of SARS‐CoV‐2 and progeny viruses (Figure 1F), recapitulating authentic viral replication. This established SARS‐CoV‐2 trVLP as a reliable model system for studying the egress pathway of SARS‐CoV‐2.

In this study, we specifically examined the egress pathway of SARS‐CoV‐2 using scanning electron microscopy (SEM) and pharmacological inhibition assays. Rab7, a member of the Ras superfamily of small Rab GTPases, was crucial for the formation and maintenance of lysosomes, and CID1067700, a competitive inhibitor of Rab7 GTPase^19^, strongly inhibited MHV egress via lysosomal exocytosis^12^. In this study, CID1067700 treatment significantly reduced lysosome formation in Caco‐2‐N cells (Figure 1C,D) and decreased intracellular LAMP1 levels in SARS‐CoV‐2 trVLP‐infected cells (Figure 1E). Lysosomal exocytosis was also regulated by intracellular calcium, which can be chelated with BAPTA‐AM^12^, ^20^. Electron microscopy revealed that SARS‐CoV‐2 trVLP‐infected cells showed lysosome‐like organelles containing multiple virions (Figure 1F, middle upper), consistent with lysosomal morphology and physiology. The results indicated that SARS‐CoV‐2 may utilize lysosomal exocytosis for egress. When lysosomal exocytosis was inhibited by CID1067700 and BAPTA‐AM, few dilated Golgi cisternae containing virions were observed (Figure 1F), suggesting that the biosynthetic secretory pathway may be a less efficient route for viral egress.

To further evaluate whether SARS‐CoV‐2 exploits the biosynthetic secretory pathway for egress, we used Brefeldin A (BFA), a small molecule that rapidly disrupts anterograde biosynthetic secretory trafficking from the endoplasmic reticulum/endoplasmic reticulum–Golgi intermediate compartment (ER/ERGIC) to the plasma membrane. The clear vacuoles densely packed with virions in BFA‐treated cells (Figure 1F) likely represented lysosome‐like vesicles, indicating that BFA treatment did not significantly affect SARS‐CoV‐2 egress.

To distinguish between viral egress and uptake pathways, the endocytosis inhibitor Dyngo‐4a was applied at 6 hpi to selectively block the uptake pathway^12^, ^21^. The dense multivesicular bodies (MVBs) with fewer virions and amorphous material (Figure 1F) likely represented intermediates in the formation of lysosome‐like vesicles involved in virion transport. MVBs were intermediates in lysosome biogenesis^22^, and it has been proposed that β‐coronaviruses may utilize MVBs for virion trafficking to lysosomes^12^. This supported the hypothesis that SARS‐CoV‐2 used multiple egress pathways, with lysosomal exocytosis as the primary mechanism and the biosynthetic pathway as a secondary, less efficient route.

### SARS‐CoV‐2 primarily uses lysosomal exocytosis for egress

**Figure 2:**
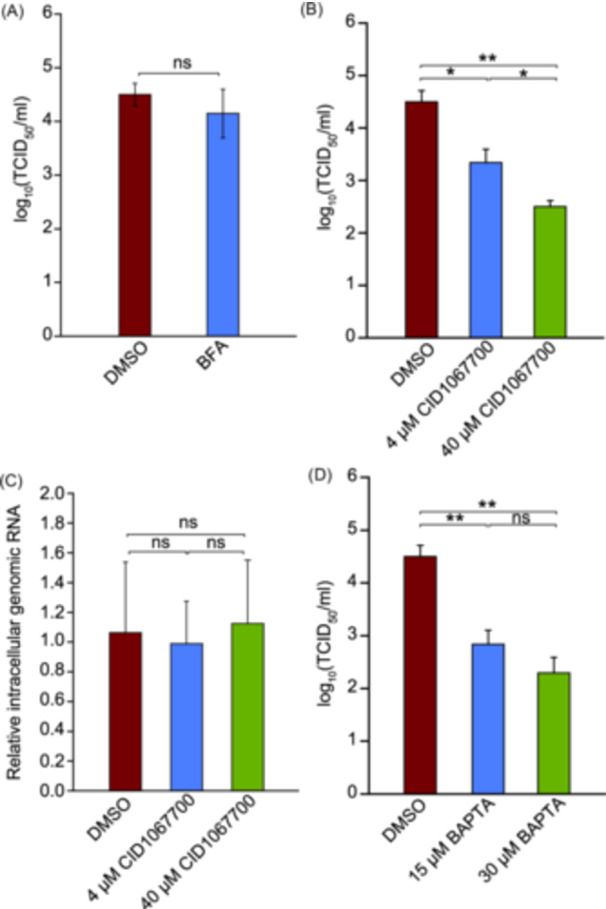
SARS‐CoV‐2 primarily uses lysosomal exocytosis for egress. (A) Effect of BFA on SARS‐CoV‐2 trVLP egress. The extracellular infectious SARS‐CoV‐2 trVLP levels in DMSO‐ and BFA‐treated cells (8–14 hpi) were quantified by the TCID_50_ assay. (B) Effects of CID1067700 on the egress of SARS‐CoV‐2 trVLP by the TCID_50_ assay. The levels of extracellular infectious SARS‐CoV‐2 trVLP in DMSO‐ and CID1067700‐treated (4 or 40 µM) SARS‐CoV‐2 trVLP‐infected cells at 8–14 hpi were quantified by the TCID_50_ assay. (C) Effects of CID1067700 on the genomic replication of SARS‐CoV‐2. Intracellular SARS‐CoV‐2 genomic RNA levels in DMSO‐ and CID1067700‐treated (4 or 40 µM) SARS‐CoV‐2‐infected cells at 8–14 hpi were quantified by relative quantitative RT‐PCR analysis. SARS‐CoV‐2 genomic RNA levels were plotted as the relative *nsp12* copy number, normalized to the housekeeping gene *GAPDH*. (D) Effects of BAPTA‐AM on the egress of SARS‐CoV‐2 trVLP quantified by the TCID_50_ assay. The levels of extracellular infectious SARS‐CoV‐2 trVLP in DMSO‐ and BAPTA‐AM‐treated (15 or 30 µM) SARS‐CoV‐2 trVLP‐infected cells at 8–14 hpi were quantified by the TCID_50_ assay. Data are presented as mean ± SD from three independent experiments. Significance was assessed using an unpaired two‐tailed *t*‐test (A) or one‐way ANOVA (B–D). ns, not significant; *p < 0.05; and **p < 0.01.

To quantify the effect of exocytosis inhibitors on the egress pathway of SARS‐CoV‐2, we used tissue‐culture infectious dose 50% (TCID_50_) assays and quantitative RT‐PCR (qRT‐PCR) to measure the infectious progeny virions and genomic RNA of SARS‐CoV‐2, respectively. TCID_50_ assays demonstrated that BFA treatment had no significant impact on SARS‐CoV‐2 trVLP egress (Figure 2A), indicating that the biosynthetic secretory pathway was not the primary route for SARS‐CoV‐2 egress. In addition, CID1067700 significantly reduced SARS‐CoV‐2 trVLP egress (Figures 2B and S1A,B), confirming that lysosomal exocytosis is the predominant egress mechanism. CID1067700 had no significant effect on SARS‐CoV‐2 genomic replication (Figures 2C and S1C), suggesting that lysosomal exocytosis is not essential for SARS‐CoV‐2 genomic replication.

TCID_50_ and qRT‐PCR assays also demonstrated that BAPTA‐AM significantly decreased SARS‐CoV‐2 trVLP egress (Figures 2D and S1D,E). Furthermore, BAPTA‐AM treatment also restricted SARS‐CoV‐2 genomic replication (Figure S1F,G). The observed differences in the effects of BAPTA‐AM and CID1067700 could be attributed to the pivotal role that intracellular calcium plays in maintaining ER functionality. Given that SARS‐CoV‐2 genomic replication occurred within replication organelles originating from the ER^23^, disrupting replication organelle function with BAPTA‐AM would likely impede SARS‐CoV‐2 genomic replication. Collectively, these results supported the notion that SARS‐CoV‐2 primarily uses lysosomal exocytosis for egress.

### SARS‐CoV‐2 trVLP infection promotes lysosomal exocytosis

**Figure 3:**
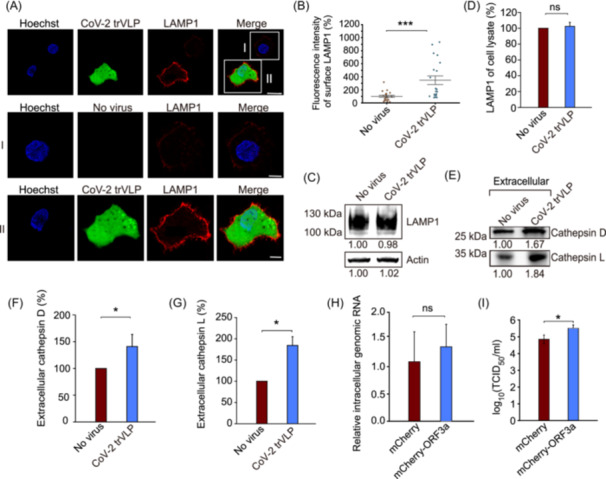
SARS‐CoV‐2 infection promotes lysosomal exocytosis via ORF3a. (A) Effect of SARS‐CoV‐2 trVLP infection on LAMP1 (red) localization at the cell surface. Representative confocal microscopy images show LAMP1 (red) localization at the cell surface in both uninfected and SARS‐CoV‐2 trVLP‐infected Caco‐2‐N cells. Plasma membrane LAMP1 was immunostained with the human LAMP‑1/CD107a lumenal domain (extracellular epitope). Magnified views of the boxed regions are shown in the lower panels (I and II). Scale bars, 25 µm for the upper image and 10 µm for the lower image (I and II). (B) Quantitative analysis of LAMP1 expression at the cell surface in both uninfected and SARS‐CoV‐2 trVLP‐infected Caco‐2‐N cells (*n* = 20 cells/group). Cells were selected from random microscopic fields, and analyses were performed under blinded conditions. The fluorescence intensity of LAMP1 at the cell surface in uninfected Caco‐2‐N cells, serving as the control group, was normalized to 100%. (C) Effect of SARS‐CoV‐2 trVLP infection on the total LAMP1 level. Representative immunoblot analyses of LAMP1 and actin in the cell lysate of uninfected and SARS‐CoV‐2 trVLP‐infected Caco‐2‐N cells are shown. The levels of LAMP1 and actin in uninfected control cells were both set to 1.0. (D) Quantitative analysis of LAMP1 levels in uninfected and SARS‐CoV‐2 trVLP‐infected Caco‐2‐N cells. The level of LAMP1 was first normalized to *actin*. The level of LAMP1 in uninfected Caco‐2‐N cells, serving as the control group, was then normalized to 100%. (E) Effects of SARS‐CoV‐2 trVLP infection on the release of cathepsin D and cathepsin L. Representative immunoblot analyses of lysosomal cathepsin D and cathepsin L released into the culture medium of uninfected and SARS‐CoV‐2 trVLP‐infected cells are shown. The levels of cathepsin D and cathepsin L in control cells were both set to 1.0. (F, G) Quantitative analysis of cathepsin D (F) and cathepsin L (G) levels in the culture medium of uninfected and SARS‐CoV‐2 trVLP‐infected cells. The levels of cathepsin D and cathepsin L were first normalized to *actin*. The levels of cathepsin D and cathepsin L in the culture medium of uninfected, serving as the control group, were then normalized to 100%. (H) Effect of SARS‐CoV‐2 ORF3a protein on the genomic replication of SARS‐CoV‐2 trVLP. Intracellular SARS‐CoV‐2 genomic RNA levels in SARS‐CoV‐2 trVLP‐infected mCherry‐expressing and mCherry‐ORF3a‐expressing cells were quantified by relative quantitative RT‐PCR analysis. SARS‐CoV‐2 genomic RNA levels were plotted as the relative *nsp12* copy number, normalized to the housekeeping gene *GAPDH*. (I) Effect of SARS‐CoV‐2 ORF3a protein on SARS‐CoV‐2 trVLP egress. The extracellular infectious SARS‐CoV‐2 trVLP levels in SARS‐CoV‐2 trVLP‐infected mCherry‐expressing and mCherry‐ORF3a‐expressing cells were quantified by the TCID_50_ assay. Mean levels ± SD from three independent experiments were plotted. Significance was assessed using an unpaired two‐tailed *t*‐test (B, D, and F–I). ns, not significant; *p < 0.05; and ***p < 0.001.

Lysosomal exocytosis is a process by which lysosomes fuse with the plasma membrane, resulting in the release of the lysosomal contents^24^. Thus, lysosomal exocytosis leads to the transfer of lysosomal membrane proteins to the plasma membrane and the secretion of lysosomal proteases to the extracellular medium^25^. In this study, we initially evaluated the effects of SARS‐CoV‐2 trVLP infection on the plasma membrane localization of the lysosomal membrane protein LAMP1. Immunofluorescence of plasma membrane LAMP1 (extracellular epitope) revealed a significant increase in plasma membrane localization of LAMP1 in SARS‐CoV‐2 trVLP‐infected cells as compared with that in uninfected cells (Figure 3A). Quantitative analysis of the LAMP1 level further supported these observations, as shown by fluorescence microscopy (Figure 3B), whereas the total levels of LAMP1 protein were not significantly different between the SARS‐CoV‐2 trVLP‐infected and uninfected cells (Figure 3C,D). These results indicated that the increased level of plasma membrane‐localized LAMP1 in SARS‐CoV‐2 trVLP‐infected cells was not due to the increase of the total level of LAMP1, but attributed to the virus‐mediated promotion of lysosomal exocytosis, leading to enhanced plasma membrane localization of LAMP1.

To further examine the effects of SARS‐CoV‐2 infection on lysosomal exocytosis, we tested the viral infection's impact on the release of lysosomal proteases. Cathepsins, including cathepsin D and cathepsin L, are essential for the maintenance of lysosome functions^26^, ^27^. As shown in Figure 3E, the levels of both cathepsin D and cathepsin L were found to be significantly increased in the extracellular medium from SARS‐CoV‐2 trVLP‐infected cells compared with those from uninfected cells. Quantitative analysis of the expression levels of cathepsin D and cathepsin L further confirmed these (Figure 3F,G). The intracellular levels of cathepsin D and cathepsin L were not significantly different between the SARS‐CoV‐2 trVLP‐infected and uninfected cells (Figure S2A). These results indicated that the increased levels of both extracellular cathepsin D and cathepsin L were not due to the increase in the level of cathepsin D and cathepsin L in SARS‐CoV‐2 trVLP‐infected cells, but attributed to the virus‐mediated promotion of lysosomal exocytosis, leading to the release of lysosomal proteases. Collectively, these findings suggest that SARS‐CoV‐2 trVLP infection promotes lysosomal exocytosis.

### SARS‐CoV‐2 ORF3a protein promotes SARS‐CoV‐2 egress

A previous study showed that ORF3a protein further facilitated MHV egress^28^. We next investigated the impact of the SARS‐CoV‐2 ORF3a protein on both genomic replication of SARS‐CoV‐2 trVLP and viral egress. Caco‐2‐N cells were transfected with mCherry‐ORF3a and subsequently infected with SARS‐CoV‐2 trVLP (Figure S2B,C). The extracellular progeny viruses and the intracellular genomic RNA were quantified by TCID_50_ and relative quantitative RT‐PCR. Figure 3H demonstrated that ORF3a expression did not significantly alter viral genomic replication. However, Figures 3I and S2D revealed a notable enhancement in the extracellular release of progeny viruses, signifying that the ORF3a protein facilitated SARS‐CoV‐2 trVLP egress. Further, absolute quantitative RT‐PCR analyses (Figure S2E,F) corroborated these findings, reinforcing the role of ORF3a in promoting viral egress. Thus, expression of SARS‐CoV‐2 ORF3a facilitated the release of SARS‐CoV‐2 trVLP into the extracellular environment.

### Lysosome deacidification facilitates SARS‐CoV‐2 egress

**Figure 4:**
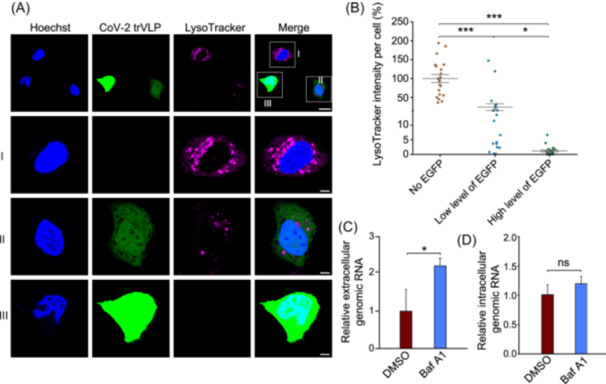
Lysosome deacidification facilitates SARS‐CoV‐2 egress. (A) Effect of SARS‐CoV‐2 trVLP infection on lysosome deacidification, measured by the fluorescence intensity of LysoTracker. Representative confocal microscopy images show LysoTracker staining in SARS‐CoV‐2 trVLP‐infected Caco‐2‐N cells at 24 hpi. The progress of the late phase of virus infection can be indicated by the GFP fluorescence intensity, where GFP serves as a marker for SARS‐CoV‐2 trVLP infection. Magnified views of the boxed regions are shown in the lower panels (I, II, and III). Scale bars, 20 µm for the upper image and 5 µm for the lower image (I, II, and III). (B) Quantitative analysis of the fluorescence intensity of LysoTracker in SARS‐CoV‐2 trVLP‐infected Caco‐2‐N cells (*n* = 20 cells/group). Cells were selected from random microscopic fields, and analyses were performed under blinded conditions. The fluorescence intensity of LysoTracker in uninfected Caco‐2‐N cells, serving as the control group, was normalized to 100%. The effect of SARS‐CoV‐2 trVLP infection on the fluorescence intensity of LysoTracker, indicative of lysosomal pH, was compared with this control. To assess the correlation between the progress of viral infection and lysosomal acidification, cells were categorized based on enhanced GFP (EGFP) intensity into two groups: those with low levels of EGFP and those with high levels of EGFP. (C, D) Effects of further lysosome deacidification on the genomic replication and egress of SARS‐CoV‐2 trVLP. Further lysosome deacidification is trigged by bafilomycin A1 (baf A1). Extracellular (C) and intracellular (D) SARS‐CoV‐2 genomic RNA levels in DMSO‐ and baf A1‐treated (10 nM) SARS‐CoV‐2 trVLP‐infected cells at 8–14 hpi were quantified by relative quantitative RT‐PCR analysis. SARS‐CoV‐2 genomic RNA levels were plotted as the relative *nsp12* copy number, normalized to the housekeeping gene *GAPDH*. Mean levels ± SD from three independent experiments were plotted. Significance was assessed using one‐way ANOVA (B) or an unpaired two‐tailed *t*‐test (C, D). ns, not significant; *p < 0.05; and ***p < 0.001.

To measure the potential effects of SARS‐CoV‐2 trVLP infection on lysosomes, we used LysoTracker dyes to measure the pH of acidic organelles, which are predominantly lysosomes. These fluorescent acidotropic probes, effectively used in studies examining the biosynthesis and acidification of lysosomes, were utilized to measure changes in lysosomal pH^12^, ^29^, ^30^. The LysoTracker dyes could efficiently label lysosomes (Figure S3A). Caco‐2‐N cells were infected with recombinant SARS‐CoV‐2 trVLP for 24 h, followed by labeling with LysoTracker Deep Red for imaging, as depicted in Figure 4A. By substituting the viral N protein in the SARS‐CoV‐2 genome with a GFP reporter, we ensured that GFP transcription and translation kinetics paralleled those of the virus's other structural and accessory proteins, thus allowing GFP fluorescence intensity to serve as an indicator of the virus infection's progression to its late phase^17^. We observed a decrease in LysoTracker fluorescence intensity concomitant with an increase in GFP fluorescence intensity (Figure 4A,B), signifying an elevation in the pH of acidic organelles, primarily lysosomes, as the SARS‐CoV‐2 trVLP infection advanced to its late phase. These findings indicated that as SARS‐CoV‐2 trVLP infection progressed, the pH of acidic organelles increased, with a corresponding increase in the pH of lysosomes, potentially promoting viral egress.

Previous research has demonstrated that a high concentration of bafilomycin A1 (100 nM) led to lysosome deacidification and disrupted autophagic flux, crucial for autophagic degradation activity^31^, while a lower concentration (10 nM) primarily affected lysosome deacidification, with minimal impact on autophagic flux^32^ (Figure S3B–D). In this study, a low concentration of bafilomycin A1 (10 nM) was added to the cells at 8–14 hpi. The total duration of the infection was 14 h. We found that lysosome deacidification by bafilomycin A1 significantly increased SARS‐CoV‐2 trVLP egress, but had no significant effect on SARS‐CoV‐2 genomic replication (Figures 4C,D and S3E). Further validation through absolute quantitative RT‐PCR analysis of SARS‐CoV‐2 genomic RNA corroborated these observations (Figure S3F,G). These findings demonstrated that SARS‐CoV‐2 trVLP infection resulted in lysosome deacidification and that further deacidification triggered by bafilomycin A1 further facilitated viral egress.

### SARS‐CoV‐2 viral proteins regulates the pH of lysosomes at different stages of viral infection

**Figure 5:**
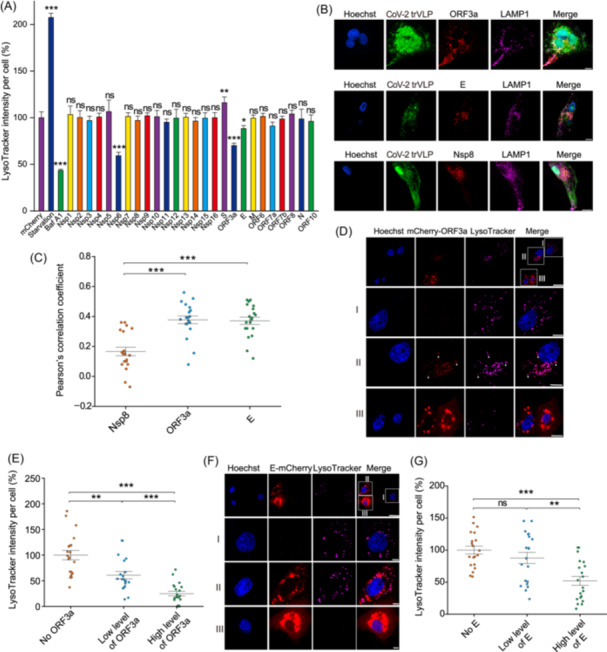
Evaluation of the effects of SARS‐CoV‐2 viral proteins on lysosome acidification. (A) Effects of SARS‐CoV‐2 viral proteins on lysosomal pH using flow cytometry analysis. All SARS‐CoV‐2 viral proteins were cloned into an mCherry‐tagged expression vector, and the mean fluorescence intensity of LysoTracker in Vero E6 cells expressing the various SARS‐CoV‐2 viral proteins was quantitatively analyzed. Cells treated with baf A1 were used as a positive control and cells treated with DMEM with no serum (starvation) were used as a negative control. The fluorescence intensity of LysoTracker in mCherry‐transfected Vero E6 cells, serving as the control group, was normalized to 100%. Mean levels ± SD from experiments performed in triplicate were plotted. (B) SARS‐CoV‐2 ORF3a and E proteins colocalized with lysosomes. Representation confocal microscopy images of Nsp8, ORF3a, and E colocalized with lysosome marker LAMP1 during SARS‐CoV‐2 infection are shown. Nsp8 was used as the control in this experiment. Scale bars, 10 µm for the upper and middle images, and 25 µm for the lower image. (C) Pearson's colocalization coefficient (PCC) quantification of lysosome marker LAMP1 with the indicated SARS‐CoV‐2 viral proteins (*n* = 20 cells/group). (D) Evaluation of the effect of ORF3a protein expression of SARS‐CoV‐2 on lysosomal pH using confocal microscopy. Representative images show LysoTracker (magenta) and Hoechst (blue) staining in mCherry‐ORF3a‐expressing Vero E6 cells. Magnified views of the boxed regions are shown in the lower panels (I, II, and III). Scale bars, 25 µm for the upper image and 10 µm for the lower image (I, II and III). (E) Quantitative analysis of LysoTracker fluorescence intensity in Vero E6 cells expressing SARS‐CoV‐2 ORF3a in (D) (*n* = 20 cells/group). The fluorescence intensity of LysoTracker in mock‐transfected Vero E6 cells, serving as the control group, was normalized to 100%. The cells were divided into two groups based on the ORF3a‐mCherry intensity: those with low levels of ORF3a and those with high levels of ORF3a. (F) Evaluation of the effect of E protein expression of SARS‐CoV‐2 on lysosomal pH using confocal microscopy. Representative images of LysoTracker (magenta) and Hoechst (blue) staining in E‐mCherry‐expressing Vero E6 cells are shown. Magnified views of the boxed regions are shown in the lower panels (I, II, and III). Scale bars, 25 µm for the upper image and 10 µm for the lower image (Ⅰ, Ⅱ and III). (G) Quantitative analysis of fluorescence intensity of LysoTracker in cells expressing SARS‐CoV‐2 E‐mCherry in (F) (*n* = 20 cells/group). The fluorescence intensity of LysoTracker in mock‐transfected Vero E6 cells, serving as the control group, was normalized to 100%. The cells were divided into two groups based on the E‐mCherry intensity: those with low levels of E and those with high levels of E. Cells were selected from random microscopic fields, and analyses were performed under blinded conditions (C, E, and G). Significance was assessed using one‐way ANOVA (A, C, E, and G). ns, not significant; *p < 0.05; **p < 0.01; and ***p < 0.001.

To elucidate the specific mechanisms through which SARS‐CoV‐2 viral proteins modulate lysosomal function to aid in viral replication, it was critical to systematically examine the impact of these proteins on lysosomal pH. All viral proteins encoded by the SARS‐CoV‐2 genome, including nonstructural, structural, and accessory proteins, were cloned into an mCherry‐tagged expression vector. This approach allowed us to investigate the effect of each viral protein on lysosomal pH using flow cytometry. The results revealed that the S protein significantly increased lysosome acidification, aligning with previous findings^33^. Conversely, Nsp6, ORF3a, and the E protein each caused a distinct level of lysosome deacidification, with Nsp6 and ORF3a showing stronger effects compared to the E protein (Figure 5A). Nsp6, a nonstructural protein expressed during the virus′s intermediate infection stage, deacidifies lysosomes and triggers pyroptosis^29^. In contrast, ORF3a, an accessory protein, and the E protein, a structural protein, are expressed later in the infection cycle and also contribute to lysosome deacidification.

We further validated the lysosome deacidification effects of ORF3a and E using confocal fluorescence imaging. In order to validate the physiological functions of ORF3a and E proteins, we conducted subcellular localization analysis of ORF3a and E proteins during viral infection (Figure 5B,C). Nsp8 was used as the control in this experiment. The analysis revealed a high Pearson's correlation coefficient between both proteins and the lysosomal marker LAMP1, indicating a high degree of colocalization. Confocal fluorescence imaging also confirmed that ORF3a and E proteins of SARS‐CoV‐2 showed substantial colocalization with lysosomes *in vitro* (Figure S4), suggesting their direct involvement in modulating lysosomal pH, which is crucial for viral egress. Quantitative analysis demonstrated that ORF3a's impact on increasing lysosomal pH was at both low expression levels and high expression levels (Figure 5D,E), whereas the E protein only increased lysosomal pH at relatively high expression levels (Figure 5F,G). These results indicated that ORF3a protein may contribute more to the virus‐mediated lysosome deacidification than E protein at the late stage of viral infection. Considering the expression of ORF3a and E proteins at the late stage of virus infection, we suggest that they might be the main viral proteins involved in lysosome deacidification to facilitate viral egress.

### The ORF3a and E proteins of SARS‐CoV‐2 inactivates lysosomal degradative activity

**Figure 6:**
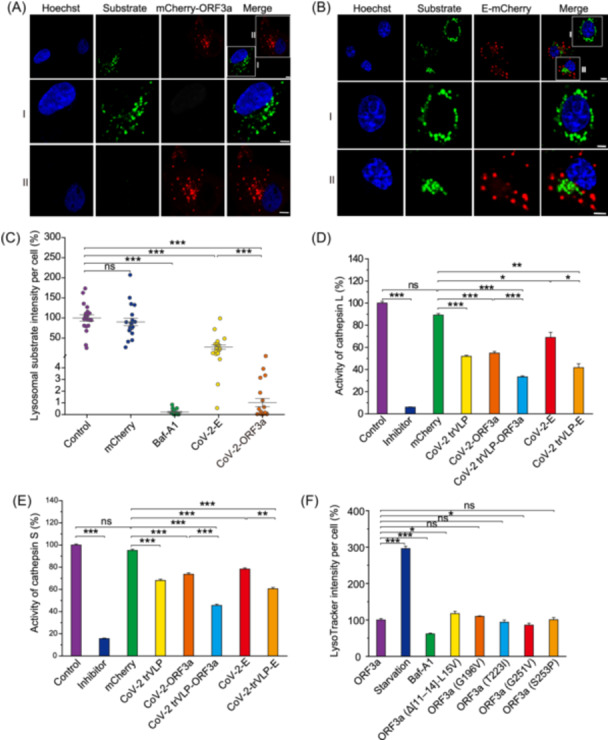
The activities of lysosomal enzymes are reduced by SARS‐CoV‐2 through ORF3a and E proteins. (A, B) Effect of the ORF3a protein (A) and E protein (B) of SARS‐CoV‐2 on lysosomal degradative activity evaluated using confocal microscopy. Representative images of cells expressing mCherry‐ORF3a or E‐mCherry treated with lysosome‐specific self‐quenched substrates are shown. The fluorescence intensity shown by the substrates was interpreted as being proportionate to the lysosomal degradative activity within the intracellular compartment. Magnified views of the boxed regions are shown in the lower panels (I, II, and III). Scale bar, 5 μm. (C) Quantitative analysis of the fluorescence intensity of lysosomal substrates in cells expressing the SARS‐CoV‐2 ORF3a or E protein using confocal microscopy (*n* = 20 cells/group). Cells were selected from random microscopic fields, and analyses were performed under blinded conditions. The fluorescence intensity of substrates in mock‐transfected Vero E6 cells, serving as the control, was normalized to 100%. The effects of ORF3a and E protein expression on lysosomal degradative activity were compared with the control. (D, E) Effects of SARS‐CoV‐2 trVLP infection, and ORF3a and E protein expression on the activities of cathepsin L (D) and cathepsin S (E). Activities were quantified using the fluorometric cathepsin L and cathepsin S activity assay kit. Cathepsin L and S activities in mock‐transfected and mock‐infected Vero‐E6‐N cells were used as controls and set to 100%. (F) Effects of naturally occurring SARS‐CoV‐2 ORF3a variants on lysosome deacidification. Quantitative analysis of the fluorescence intensity of LysoTracker in Vero E6 cells expressing naturally occurring SARS‐CoV‐2 ORF3a variants is shown. The fluorescence intensity of LysoTracker in ORF3a‐transfected Vero E6 cells, serving as the control group, was normalized to 100%. Mean levels ± SD from experiments performed in triplicate were plotted (C‐F). Significance was assessed using one‐way ANOVA (C–F). ns, not significant; *p < 0.05; **p < 0.01; and ***p < 0.001.

Next, we investigated the impact of SARS‐CoV‐2 ORF3a and E proteins on lysosomal degradative activity, pivotal for viral egress. Lysosomal enzymes operate optimally at specific pH levels, with even minor pH shifts significantly impacting lysosomal protease activity^34^, ^35^. In this study, we used lysosome‐specific self‐quenched substrates to quantify the activity of lysosomal degradation enzymes in cells expressing SARS‐CoV‐2 ORF3a or E proteins. The fluorescence intensity generated by the degradation of substrates was proportional to the lysosomal degradative activity and was quantified by fluorescence microscopy or flow cytometry. Fluorescence intensity, indicative of lysosomal degradative activity, was markedly lower in cells expressing mCherry‐ORF3a compared to control cells, as illustrated in Figure 6A. A similar decrease in fluorescence intensity was observed in E‐mCherry‐expressing cells compared to control cells (Figure 6B). Quantitative analyses further substantiated these observations, indicating a significant decrease in lysosomal degradative activity in cells expressing either ORF3a or E proteins, with ORF3a exerting a more significant effect (Figure 6C). Flow cytometry also corroborated these findings, revealing a reduction in substrate fluorescence intensity in cells expressing either ORF3a or E proteins, with a more pronounced decrease observed with ORF3a (Figure S5A). Furthermore, the proportion of cells with positive lysosomal activity was significantly reduced in the presence of ORF3a and E proteins, especially with ORF3a (Figure S5B). Collectively, our data demonstrated that the ORF3a and E proteins of SARS‐CoV‐2 notably suppressed lysosomal degradation enzyme activities, with ORF3a exerting a more significant effect.

### The activities of lysosomal cathepsins are reduced by SARS‐CoV‐2 through ORF3a and E proteins

Cathepsin S and cathepsin L played critical roles in antigen processing through the successive cleavages of the invariant chain^36^. Previous research on MHV infection highlighted how MHV disrupted antigen processing and presentation via lysosome deacidification^12^. Additionally, although cathepsin L was essential for cleaving the spike protein during endosomal entry of SARS‐CoV‐2^37^, ^38^, it would cause premature cleavage and inactivate progeny virus particles during viral egress. Infectious bronchitis coronavirus could alter Golgi pH to protect the spike protein from premature cleavage during viral egress^10^. The impact of SARS‐CoV‐2‐induced lysosome deacidification on the activities of cathepsins remains to be elucidated.

We investigated how SARS‐CoV‐2 trVLP infection and the expression of its ORF3a and E proteins affect the activities of cathepsin S and cathepsin L. Our findings, as illustrated in Figure 6D,E, revealed that SARS‐CoV‐2 trVLP infection diminished the activities of both cathepsin S and cathepsin L. Furthermore, expression of the ORF3a and E proteins independently suppressed the activities of these cathepsins, with ORF3a having a more significant impact than E proteins. Moreover, in SARS‐CoV‐2 trVLP‐infected cells, the ORF3a and E proteins further decreased cathepsin S and cathepsin L activities beyond the reduction caused by the virus alone. These results indicated that SARS‐CoV‐2, particularly through the ORF3a and E proteins, inhibited lysosomal cathepsin activity.

### Naturally occurring SARS‐CoV‐2 ORF3a variants affect viral pathogenesis via altered abilities to trigger lysosome deacidification

Spike protein variation of SARS‐CoV‐2 is the main factor determining the ability of the virus to infect cells and transmit between different hosts; intensive studies have focused on the continuous mutation of the *S* gene^39^, ^40^, ^41^. However, ORF3a variation may also play vital roles in the viral replication, pathogenesis, and severity of COVID‐19^42^.

In this study, we found that the fluorescence intensity of LysoTracker was significantly increased in cells expressing ORF3a(Δ[11–14]‐L15V) compared with those expressing wild‐type ORF3a, indicating a diminished capacity for lysosome deacidification (Figure 6F). This might explain why SARS‐CoV‐2 strain UF‐8, possessing this variant, showed delayed cytopathic effects and diminished viral replication^43^. A previous study also linked the ORF3a(G251V) variant to severe infection and the ORF3a(S253P) variant to fatal infection^44^. We also observed that the ORF3a(G251V) variant demonstrated an increased ability to deacidify lysosomes compared to the wild type (Figure 6F), potentially facilitating greater viral egress and contributing to more severe infections. Conversely, the ORF3a(S253P) variant and the ORF3a(G196V) variant, despite being linked to fatal infections and asymptomatic infections, respectively^45^, showed no significant effect on lysosome deacidification (Figure 6F), suggesting that they may influence other ORF3a functions. Furthermore, our findings indicated that the ORF3a(T223I) variant did not significantly impact lysosome deacidification (Figure 6F). However, it has been previously demonstrated that this mutation impairs lipid droplet accumulation, which may contribute to the reduced replication and transmission observed in Omicron variants^46^, ^47^. Taken together, these results suggest that variations in the ORF3a protein differentially may affect SARS‐CoV‐2's lifecycle through altered lysosome deacidification capabilities, thereby influencing viral egress and associated pathogenic outcomes.

## DISCUSSION

The egress pathway of SARS‐CoV‐2 remains controversial^12^, ^14^, ^16^. One study, utilizing transmission electron microscopy (TEM) and immunofluorescence staining, found SARS‐CoV‐2 virus particles within lysosome‐like and LAMP1‐positive organelles, suggesting lysosomal exocytosis as the egress pathway^12^. However, subsequent studies, also using TEM, identified SARS‐CoV‐2 release via small secretory vesicles, proposing the secretory pathway as the egress mechanism^15^. Upon comparing these studies, we noted that the first study performed ultrastructural characterizations at 24 hpi^12^, whereas the second study observed cells at 10 hpi^15^. This temporal difference in pathway utilization may partially explain the observed discrepancies, suggesting that SARS‐CoV‐2 could utilize both egress pathways at different stages of infection or utilize different pathways with varying efficiencies. In this study, we used biochemical inhibitors BFA, Dyngo‐4a, CID1067700, and BAPTA‐AM in combination with SEM and TCID_50_ to precisely delineate the primary egress pathway of SARS‐CoV‐2 trVLP. The vesicular structures in the SEM images were heterogeneous (Figure 1F). Specifically, numerous vacuoles densely packed with virions in untreated, BFA‐treated, and Dyngo‐4a‐treated cells likely represented lysosomes, suggesting that SARS‐CoV‐2 predominantly utilized lysosomal exocytosis for viral egress. Additionally, few dilated Golgi cisternae containing virions suggested viral egress via the biosynthetic secretory pathway, particularly when the lysosomal exocytosis pathway was inhibited by CID1067700 and BAPTA‐AM. The TCID_50_ assay and pharmacological inhibition assays also demonstrated that SARS‐CoV‐2 primarily utilized lysosomal exocytosis for viral egress. Based on TCID_50_ fold‐differences, we estimated that about 2.2% of progeny virions were released via the biosynthetic pathway, compared to lysosomal exocytosis. As these routes may differ in sensitivity to pharmacological inhibition, this value should be considered an estimate. These findings substantiate the hypothesis that SARS‐CoV‐2 utilizes multiple egress mechanisms, predominantly relying on lysosomal exocytosis, while also engaging the biosynthetic secretory pathway as a supplementary route with reduced efficiency.

It has been shown that MHV used lysosomal exocytosis for egress, and MHV infection further promoted lysosomal exocytosis, which may facilitate viral egress^12^. Our study showed that inhibition of lysosomal exocytosis blocked SARS‐CoV‐2 trVLP egress, indicating that SARS‐CoV‐2 also utilized lysosomal exocytosis for the release of progeny viruses. However, whether SARS‐CoV‐2 infection can further promote lysosomal exocytosis remains unclear. In this study, we demonstrated that SARS‐CoV‐2 trVLP infection enhanced lysosomal exocytosis. Specifically, SARS‐CoV‐2 trVLP infection promoted both the localization of lysosomal membrane proteins to the plasma membrane and boosted the secretion of lysosomal cathepsins into the extracellular environment (Figure 3A–G). Previous research has indicated the role of the ORF3a protein in enhancing lysosomal exocytosis^28^. MHV, despite lacking an ORF3a homolog, utilized lysosomal exocytosis for egress^52^, and co‐expression of the ORF3a protein of SARS‐CoV‐2 further facilitated MHV egress^28^. Nevertheless, whether ORF3a influences SARS‐CoV‐2 trVLP infection remains to be elucidated. Our study demonstrated that while the ORF3a protein of SARS‐CoV‐2 did not significantly impact viral genome replication (Figure 3H), it notably enhanced the extracellular release of progeny virions (Figure 3I). Our results indicated that SARS‐CoV‐2 promoted lysosomal exocytosis via ORF3a to facilitate viral egress^28^.

Coronaviruses using lysosomal exocytosis for their release modulated lysosomal pH, leading to either acidification or deacidification^12^, ^13^. In this study, we used SARS‐CoV‐2 trVLPs, which utilized GFP fluorescence intensity as an indicator of late‐phase viral infection progression^17^, to examine the lysosomal pH alterations induced by SARS‐CoV‐2. Our findings indicated a significant increase in lysosome deacidification during the late phase of infection, suggesting that the expression of SARS‐CoV‐2 viral proteins may trigger lysosome deacidification. Furthermore, our data revealed that bafilomycin A1‐induced lysosome deacidification significantly enhanced SARS‐CoV‐2 egress, underscoring the specific role of lysosome deacidification in viral release mechanisms.

The alterations in lysosomal pH induced by viral proteins led to changes in the activity of lysosomal enzymes, potentially affecting viral replication^35^. The effects of multiple SARS‐CoV‐2 viral proteins on lysosomal pH have previously been investigated, but the results for ORF3a and E proteins, in particular, have been inconsistent and thus remain controversial^12^, ^30^, ^48^, ^49^, ^50^, ^51^, ^52^. While some studies have shown that the SARS‐CoV‐2 E protein deacidifies the lysosomes^48^, ^49^, ^50^, ^52^, another study showed that the E protein had no effect on the pH of lysosomes^51^. In addition, while some studies have shown that the SARS‐CoV‐2 ORF3a protein deacidified the lysosomes^12^, ^48^, another study showed that ORF3a protein further acidified lysosomes^30^. On the basis of the instructions for LysoTracker probes and a previous report^29^, we used a treatment with 60 nM Lysotracker Deep Red for 1 h to evaluate the impact of all SARS‐CoV‐2 viral proteins on lysosomal pH. Our results showed that the ORF3a and E proteins of SARS‐CoV‐2 could both increase the pH of lysosomes. Given the pH sensitivity of lysosomal enzymes, we hypothesized that the increased pH would inactivate them partially. This hypothesis was confirmed by our observation that the expression of ORF3a and E proteins led to a noticeable decrease in the activities of lysosomal degradation enzymes and cathepsins (Figure 5). Our study demonstrated that both the ORF3a and E proteins induced lysosome deacidification during viral egress, and bafilomycin A1 further enhanced this process by inhibiting lysosome acidification, likely boosting viral egress beyond baseline levels.

Additionally, normal lysosome pH (4.5–5.0) supported cathepsin activity, which cleaved the spike protein of SARS‐CoV‐2 virions, potentially leading to premature inactivation. Cathepsin L in the lysosomes cleaved the S2' site of spike protein during endosomal entry^37^, ^38^, ^53^, and inactivation of cathepsin L protected progeny viruses from premature cleavage during viral egress. The infectious bronchitis coronavirus modulated Golgi apparatus pH to prevent premature cleavage of the spike protein during viral egress^10^. Our study found that SARS‐CoV‐2 infection diminished the activities of cathepsin S and cathepsin L by inducing lysosome deacidification, a process mediated by the ORF3a and E proteins. The deacidification may protect progeny viruses from premature cleavage during viral egress. Therefore, we propose that lysosome deacidification is probably rate‐limiting for viral egress, and further experiments, including genetic perturbation approaches such as ORF3a and E protein knockouts, will be needed to validate these findings in a physiological context.

Key viral proteins of SARS‐CoV‐2, such as the Spike protein, were significant determinants of viral pathogenicity^54^, while accessory proteins, including the ORF3a protein, may also modulate its pathogenicity. Previous research has linked certain naturally occurring ORF3a variants to a spectrum of COVID‐19 manifestations, including cytopathic effects, viral replication rates, asymptomatic infections, severe infections, and fatal outcomes^43^, ^44^, ^45^, ^55^. In this study, we found that these natural ORF3a variants altered lysosome deacidification capabilities, shedding light on the mechanism linking ORF3a mutations with COVID‐19 symptomatology.

The mechanism underlying lysosome deacidification during SARS‐CoV‐2 egress remains poorly understood. This process may involve multiple mechanisms. First, lysosomes maintained their acidic pH gradient by utilizing the activity of proton‐pumping V‐ATPases, which actively pumped protons into the lysosome lumen to maintain its acidic environment. Some viral proteins and potentially certain host factors could directly or indirectly inhibit V‐ATPase activity, inducing lysosome deacidification^29^, ^56^. Alternatively, viroporins may function as proton leak channels, potentially contributing to lysosome deacidification^10^, ^57^. While the ORF3a‐reconstituted liposomes showed nonselective cation channel activity^58^, structural and functional experiments support the conclusion that ORF3a is not an ion channel^59^. The SARS‐CoV‐2 E protein formed a cation channel that was permeable to monovalent cations^49^, ^60^. Determining the specific roles of the ORF3a and E proteins, whether inhibiting V‐ATPase activity or functioning as proton leak ion channels, is essential for targeted therapeutic strategies, necessitating further experimental validation.

**Figure 7:**
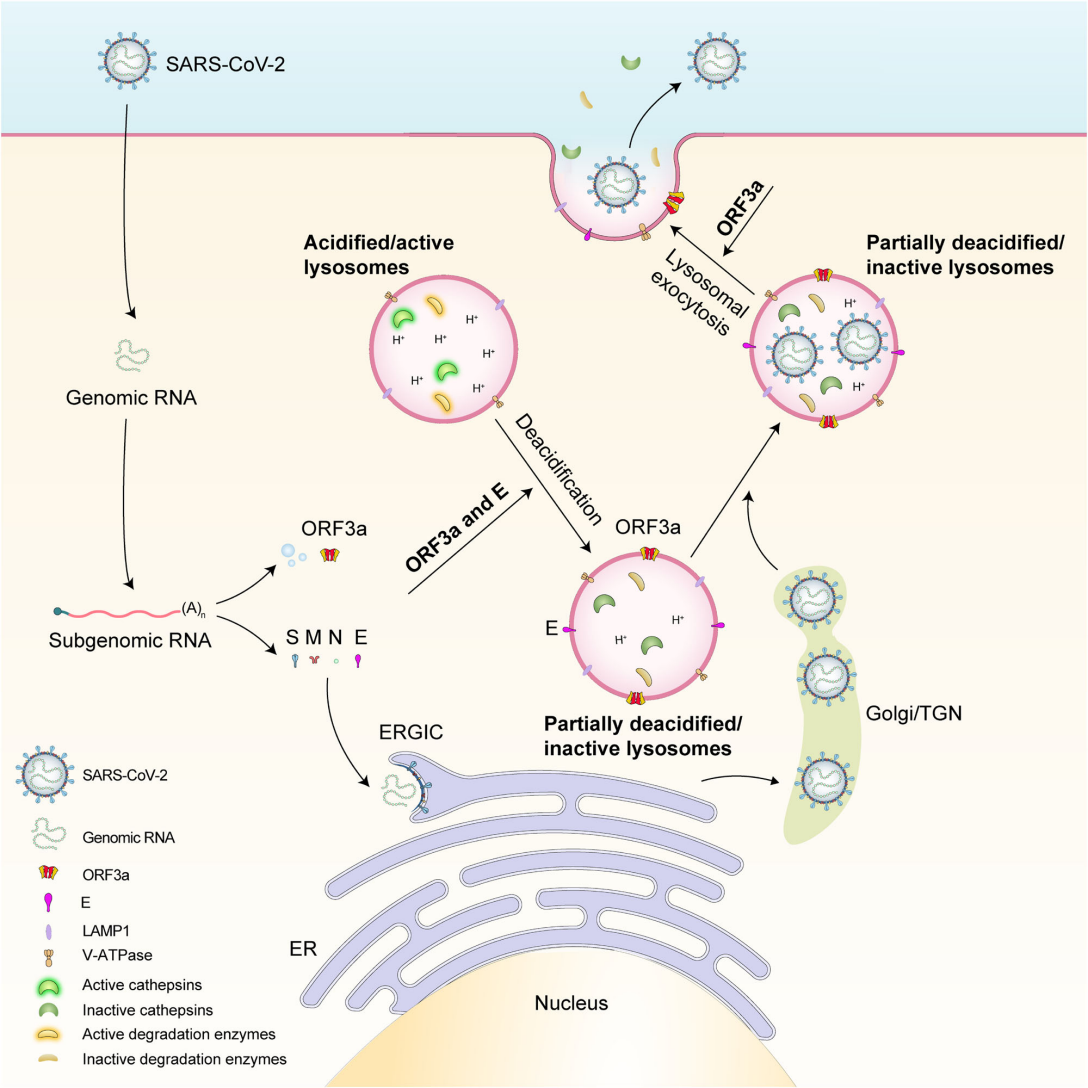
Proposed egress pathway of SARS‐CoV‐2. Assembly of progeny SARS‐CoV‐2 virions is initiated by the interaction of newly synthesized viral genomic RNA with the spike (S), membrane (M), envelope (E), and nucleocapsid (N) proteins, followed by budding into the lumen of the endoplasmic reticulum (ER) and the endoplasmic reticulum–Golgi intermediate compartment (ERGIC). The assembled virions are subsequently transported to the Golgi apparatus and trans‐Golgi network (TGN) for post‐translational modifications. In SARS‐CoV‐2, the egress of virions predominantly occurs through lysosomal exocytosis, a mechanism that is augmented by the ORF3a protein, thereby facilitating viral release. SARS‐CoV‐2 infection leads to the deacidification of lysosomes, a process mediated by the ORF3a and E proteins. This alteration in lysosomal pH impairs the enzymatic activities of cathepsins and other lysosomal degradation enzymes, potentially safeguarding nascent virions from premature proteolytic cleavage and degradation.

In summary, using drug treatments, pH‐sensitive fluorescent probes, lysosomal enzyme activity assays, and SARS‐CoV‐2 trVLPs, our findings demonstrated that the promotion of lysosomal exocytosis efficiency and lysosome deacidification were pivotal determinants of SARS‐CoV‐2 egress (Figure 7). Specifically, SARS‐CoV‐2 promoted lysosomal exocytosis through the ORF3a protein as a mechanism for viral exit, and lysosome deacidification played an essential role in facilitating viral egress. The ORF3a and E proteins of SARS‐CoV‐2 induced lysosome deacidification, leading to the inactivation of lysosomal degradation enzymes and cathepsin L, which might potentially prevent the premature cleavage of the spike proteins and degradation of the virions. The mechanism of SARS‐CoV‐2 egress revealed in this study might provide a new opportunity for developing antiviral strategies and shed light on the mechanism of other viruses that egress via lysosomal exocytosis.

## MATERIALS AND METHODS

### Cell lines and virus strains

Human embryonic kidney 293 cells that expressed a mutant version of the SV40 large T antigen (HEK293T), *Cercopithecus aethiops*‐derived epithelial kidney cells (Vero E6), Caco‐2 (human epithelial colorectal adenocarcinoma), and Caco‐2‐N cells were cultured in Dulbecco's modified Eagle medium supplemented with 10% (vol/vol) fetal bovine serum (FBS), 100 U/ml penicillin, and 100 mg/ml streptomycin. All cells were cultured in a 5% (vol/vol) CO_2_ and 90% humidity incubator at 37°C. Each cell line was tested to ensure that it was free from mycoplasma before being used in experiments. The cell lines and virus strains used in this study are detailed in Table S1.

### Cloning of expression constructs

The following SARS‐CoV‐2 genes were cloned into pcDNA3.1‐mCherry using the NEBuilder HiFi DNA Assembly: *Nsp1*, *Nsp2*, *Nsp3*, *Nsp4*, *Nsp5*, *Nsp6*, *Nsp7*, *Nsp8*, *Nsp9*, *Nsp10*, *Nsp11*, *Nsp12*, *Nsp13*, *Nsp14*, *Nsp15*, *Nsp16*, *S*, *ORF3a*, *E*, *M*, *ORF6*, *ORF7a*, *ORF7b*, *ORF8*, *N*, *ORF10*, and *LAMP1*. ORF3a(∆[11–14]‐L15V), ORF3a(G196V), ORF3a(T223I), ORF3a(G251V), and ORF3a(S253P) were constructed using pcDNA3.1‐mCherry‐ORF3a. The plasmids used in this study are detailed in Table S1.

### Virus infection

Lentivirus packaging of vesicular stomatitis virus G protein (VSV‐G) pseudotyped lentiviruses and construction of Caco‐2‐N cells that expressed SARS‐CoV‐2 N protein were performed as described elsewhere^18^. Since the Caco‐2‐N cells were not quite stable and the N protein expression in Caco‐2‐N cells could be rapidly decreased after several passages, Caco‐2‐N cells needed to be re‐transduced to maintain the high expression of SARS‐CoV‐2 N protein before virus infection. SARS‐CoV‐2 trVLP titration was determined using a TCID_50_ endpoint dilution method as described elsewhere^18^. To amplify the virus, Caco‐2‐N cells were infected with SARS‐CoV‐2 trVLPs at an MOI of 0.1 for 2 h, washed three times in PBS, and subsequently incubated in low‐serum DMEM supplemented with 3% FBS. After 48 hpi, supernatants were harvested and centrifuged at 500 g for 5 min to remove cell debris. The collected SARS‐CoV‐2 trVLPs were further concentrated via polyethylene glycol 6000 (PEG‐6000) precipitation as described elsewhere^61^. Virus pellets were resuspended in Tris–NaCl–EDTA (TNE) buffer supplemented with 1% BSA, quantified by TCID_50_ ^62^, aliquoted, and stored at ‐80°C until use as previously described^63^.

To investigate the effect of drugs on SARS‐CoV‐2 genomic replication and egress, Caco‐2‐N cells were infected at an MOI of 1 for 2 h, and thoroughly washed three times with PBS. All the virus particles that were not internalized were removed by incubation with trypsin for 2 min, followed by neutralization with DMEM with 10% FBS. Drugs were added to cells 8 hpi and maintained to 14 hpi. Cell lysates and supernatants were collected for the following RNA extraction and quantitative PCR analysis. The Shenzhen Institute of Synthetic Biology, Shenzhen Institutes of Advanced Technology, Chinese Academy of Sciences approved the SARS‐CoV‐2 trVLP trans‐complementation system in BSL‐2. All the experiments in this study involving SARS‐CoV‐2 trVLP infections were performed in BSL‐2 following the regulations. The chemicals used in this study are detailed in Table S1.

### Electron microscopy

Cryo‐TEM samples were prepared using a Vitrobot IV at 10°C with 100% humidity. Carbon film‐coated 300‐mesh copper grids (Quantifoil® R 1.2/1.3 300 Mesh, Cu) were rendered hydrophilic via glow discharge using a Pelco EasiGlow Glow Discharge. Subsequently, a 3 μl sample was applied to each grid, adsorbed for 10 s, and blotted for 3.5 s with a Ted Pella filter paper before rapid immersion in liquid ethane cooled by liquid nitrogen. The grids were stored in liquid nitrogen until imaging on a Tundra TEM at 100 kV with an electron dose of 38 electrons/Å.

For conventional SEM analysis, cells were fixed overnight at 4°C with 2.5% glutaraldehyde in 0.1 M PBS (pH 7.4), rinsed thrice with PBS, and stained with 1% osmium tetroxide and 0.8% potassium ferricyanide for 2 h at 4°C. After washing with distilled water, samples were subjected to en‐bloc staining in 1% uranyl acetate at 4°C overnight. Samples were dehydrated with graded ethanol and embedded in resin. Ultrathin sections (70 nm) were cut using a Leica UC7 ultramicrotome with a Diatome diamond knife. Post‐staining was carried out with uranyl acetate and lead citrate. Grids were examined using a Zeiss GEMINISEM 360 field emission scanning electron microscope (Germany).

### Western blot

After the specified procedure, cells were trypsinized with 0.05% Trypsin‐EDTA, rinsed 3 times with PBS, and lysed with lysis buffer supplemented with Halt™ Protease and Phosphatase Inhibitor Cocktail for 15 min on ice. The cell lysates were further centrifuged for 13,000*g* for 10 min at 4°C. To quantify the levels of cathepsin D and cathepsin L in culture medium, the same number of Caco‐2‐N cells was seeded into 6‐well cell culture plates and infected with SARS‐CoV‐2 trVLP at an MOI of 5. After 24 h, extracellular culture medium was collected at 500*g* for 10 min to remove cell debris and was then concentrated using a centrifugal concentrator at 4°C. Total protein concentrations of cell lysates and supernatants were quantified using the Pierce BCA kit. The cell lysates and supernatants were mixed with SDS‐PAGE loading buffer with β‐mercaptoethanol and boiled for 10 min. The samples were loaded on 4–12% gradient tris‐glycine gels and transferred onto PVDF membranes. The following procedures of Western blot were performed as previously described^64^. The antibodies used in this study are detailed in Table S1.

### Quantitative analysis of the pH of lysosomes by flow cytometry

Vero E6 cells were transiently transfected using Lipofectamine™ 3000 Reagent following the reagent protocol provided by the manufacturer. Quantitative analysis of the pH of lysosomes by flow cytometry was performed as previously reported^29^. Briefly, 24 h post transfection, the cells were treated with 60 nM Lysotracker Deep Red for 1 h. Cells were washed three times with PBS, trypsinized with 0.05% trypsin‐EDTA, and resuspended in 1× PBS. The cells were kept on ice and immediately analyzed on a BD Biosciences cell sorter. Cells treated with 100 nM bafilomycin A1 for 24 h were used as a positive control and cells treated with DMEM with no serum for 24 h were used as a negative control. The chemicals used in this study are detailed in Table S1.

### Immunofluorescence staining

For intracellular protein staining, cells were fixed in freshly prepared 4% paraformaldehyde (PFA) in PBS for 10 min at room temperature and blocked in freshly prepared 10% FBS in PBS for 1 h at 37°C. Primary antibodies were diluted in 10% FBS in PBS supplemented with 0.2% saponin and incubated overnight at 4°C. Secondary antibodies were diluted in 10% FBS in PBS supplemented with 0.2% saponin and incubated for 2 h at room temperature. Cells were washed with PBS three times and mounted. For plasma membrane LAMP1 staining, cells were incubated at 4°C for 20 min, human LAMP‑1/CD107a lumenal domain (extracellular epitope) antibody diluted in PBS was added, and the mixture was incubated on ice for 30 min. Cells were washed with prechilled PBS on ice, and then incubated with the corresponding chilled, diluted secondary antibody in PBS for 30 min on ice. Cells were washed with chilled PBS three times, fixed in freshly prepared chilled 2% PFA for 5 min on ice, rinsed with chilled PBS three times, and mounted. The antibodies used in this study are detailed in Table S1.

### Quantitative RT‐PCR analysis

Cell lysates and supernatants were collected and total RNA was extracted using the HiPure Total RNA Kit and the HiPure viral RNA Kit, respectively. Briefly, to quantify the copy numbers of SARS‐CoV‐2 genomic RNA, quantitative RT‐PCR analysis was executed using a HiScript® II U+ One Step qRT‐PCR Probe Kit in the Real‐Time Thermal Cyclers (Analytik Jena, qTOWER^3^). A serial dilution of the quantified plasmid (pcDNA3.1‐nsp12) was used as the standard template to draw the standard curve. The primers used in this study are detailed in Table S1. The PCR conditions were as follows: an initial cycle at 50°C for 15 min, followed by a cycle at 95°C for 30 s, and then 45 cycles of 95°C for 10 s and 60°C for 45 s each.

### Confocal light microscopy

All images were acquired on a Nikon AX R confocal microscope system using a 60× oil immersion objective lens. Cell nuclei were stained with Hoechst 33342 and excited using a 405 nm laser. The EGFP and lysosome‐specific self‐quenched substrates were excited using a 488 nm laser. LAMP1‐mCherry, ORF3a‐mCherry, E‐mCherry, and NorthernLights™ anti‐sheep IgG‐NL557 were excited using a 561 nm laser. The LysoTracker Deep Red fluorophores and Goat Anti‐Mouse IgG H&L (Alexa Fluor® 647) were excited using a 640 nm laser.

To quantify the fluorescence intensity, the DAPI, FITC, TRITC, and Cy5 channels were split by ImageJ. The fluorescence intensity of each cell was then selected by freehand selections and measured by ImageJ. LysoTracker intensity per cell (%) was calculated by dividing the LysoTracker intensity per cell of each group by the mean LysoTracker intensity per cell of the control group. About 20 cells of each group were randomly selected from 5‐10 images for analysis.

## AUTHOR CONTRIBUTIONS

**Fujun Qin**: Conceptualization; data curation; formal analysis; funding acquisition; investigation; methodology; project administration; resources; software; validation; visualization; writing—original draft; writing—review and editing. **Chuang Yan**: Investigation. **Zizheng Liu**: Investigation; methodology. **Dianbing Wang**: Funding acquisition; methodology; resources. **Huimin Zhong**: Investigation; methodology. **Qiang Ding**: Methodology; resources. **Minghai Chen**: Conceptualization; funding acquisition; methodology; resources; supervision; writing—original draft; writing—review and editing. **Xian‐En Zhang**: Conceptualization; funding acquisition; project administration; resources; supervision; writing—original draft; writing—review and editing.

## ETHICS STATEMENT

This study did not involve the use of animals or human subjects, nor did it utilize biological samples or tissues from these sources.

## CONFLICT OF INTERESTS

The authors declare no conflict of interests.

## Supporting information

Supplemental Text and Figures.

## Data Availability

All data underpinning the findings of this study are accessible within the article and its Supporting Information, or can be obtained from the corresponding authors upon reasonable request.
