# Supplementary material for: SARS‐CoV‐2 enhances lysosomal exocytosis and deacidifies lysosomes to facilitate viral release
Source: mLife. 2026 Jun 26;5(3):339–54. doi: 10.1002/mlf2.70065 (PMC13327611; doi:10.1002/mlf2.70065)
Supplement: Supplementary file 1 — Supplemental Text and Figures. [file MLF2-5-339-s001.docx]

**Supporting Information for**

**SARS-CoV-2 enhances** **lysosomal exocytosis and deacidifies lysosomes to facilitate viral release**

Fujun Qin,^1^ Chuang Yan,^1^ Zizheng Liu,^3^ Dianbing Wang,^3^ Huimin Zhong,^1^ Qiang Ding,^4^ Minghai Chen^1^ #, Xian-En Zhang^2,1,3^ #

^1^ State Key Laboratory of Quantitative Synthetic Biology, Shenzhen Institute of Synthetic Biology, Shenzhen Institutes of Advanced Technology, Chinese Academy of Sciences, Shenzhen 518055, China

^2^ Faculty of Synthetic Biology, Shenzhen University of Advanced Technology, Shenzhen, 518055, China

^3^ Key Laboratory of Biomacromolecules (CAS), National Laboratory of Biomacromolecules, CAS Center for Excellence in Biomacromolecules, Institute of Biophysics, Chinese Academy of Sciences, Beijing 100101, China

^4^ School of Medicine, Tsinghua University, Beijing100101, China

# Address correspondence to Xian-En Zhang, Email: [zhangxe@ibp.ac.cn](mailto:zhangxe@ibp.ac.cn) or Minghai Chen, Email: [mh.chen1@siat.ac.cn](mailto:mh.chen1@siat.ac.cn)

**This file includes:**

Supporting text

Figures S1 to S5

Tables S1

**Supplementary Figures**

**Figure S1**


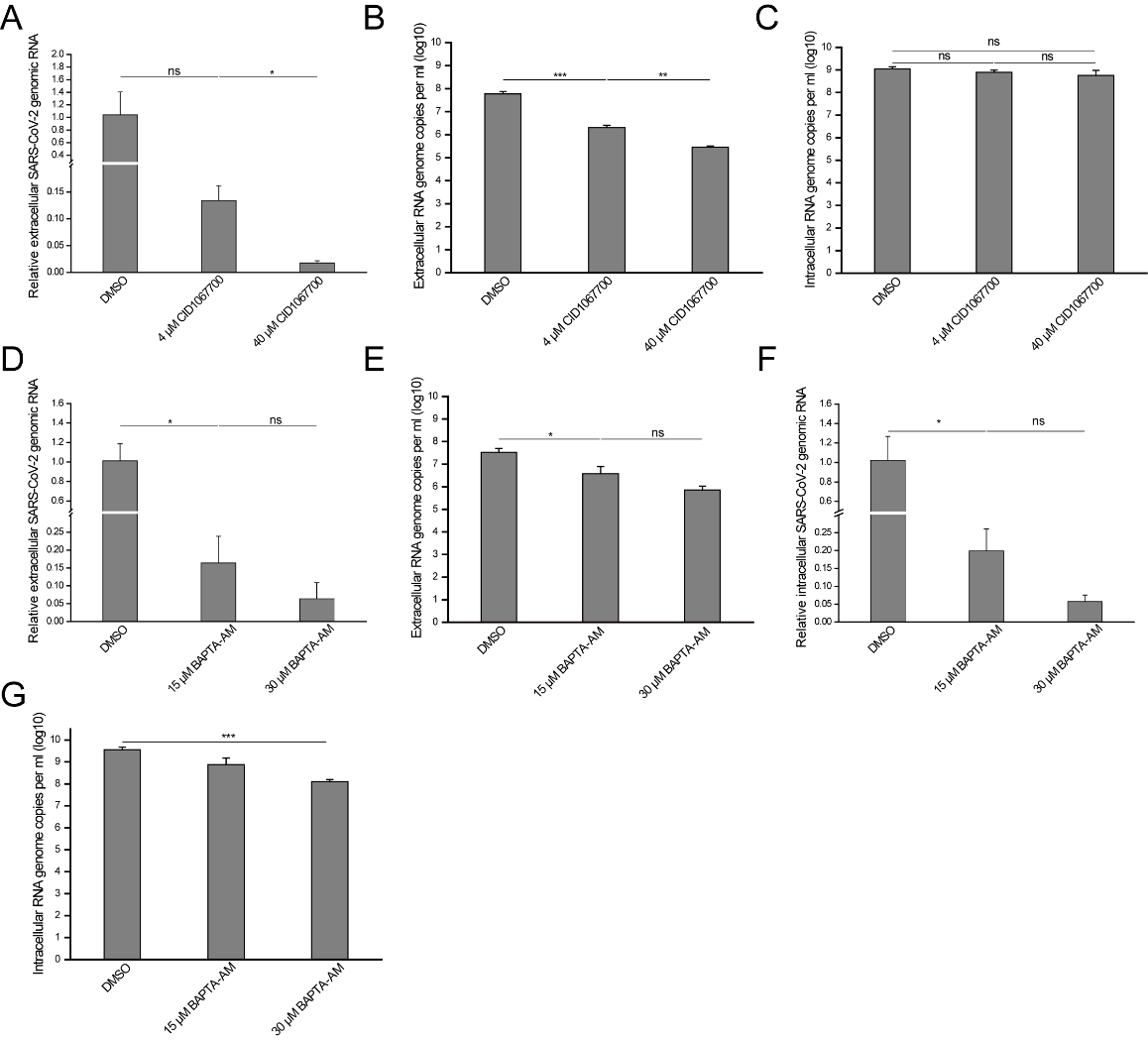


**Supplementary Figure S1.** **SARS-CoV-2 uses lysosomal exocytosis for egress. Related to Figure 2.**

(A) Effects of CID1067700 on the egress of SARS-CoV-2 trVLP by relative quantitative RT-PCR analysis. Extracellular SARS-CoV-2 genomic RNA levels in DMSO- and CID1067700-treated (4 or 40 µM) SARS-CoV-2-trVLP-infected cells at 8–14 hpi were quantified by relative quantitative RT-PCR analysis. SARS-CoV-2 genomic RNA levels were plotted as the relative Nsp12 copy number, normalized to the housekeeping gene GAPDH. Mean levels ± SD from three independent experiments were plotted. (B, C) Investigation the effects of CID1067700 on the genomic replication and egress of SARS-CoV-2 using absolute quantitative RT-PCR analysis. Extracellular (B) and intracellular (C) SARS-CoV-2 genomic RNA levels in DMSO- and CID1067700-treated (4 or 40 µM) SARS-CoV-2-infected cells at 8–14 hpi were quantified. A serial dilution of the quantified plasmid (pcDNA3.1-Nsp12) was used as standard template for generating the standard curve. Mean levels ± SD from three independent experiments were plotted. (D) Extracellular SARS-CoV-2 genomic RNA levels in DMSO- and BAPTA-AM-treated (15 or 30 µM) SARS-CoV-2-infected cells at 8–14 hpi were quantified by relative quantitative RT-PCR analysis. Mean levels ± SD from three independent experiments were plotted. (E) Effects of lysosomal exocytosis inhibitor BAPTA-AM on egress of SARS-CoV-2 trVLP using absolute quantitative RT-PCR analysis. Mean levels ± SD from three independent experiments were plotted. (F) Intracellular SARS-CoV-2 genomic RNA levels in DMSO- and BAPTA-AM-treated (15 or 30 µM) SARS-CoV-2-infected cells at 8–14 hpi were quantified by relative quantitative RT-PCR analysis. Mean levels ± SD from three independent experiments were plotted. (G) Effects of lysosomal exocytosis inhibitor BAPTA-AM on the genomic replication of SARS-CoV-2 using absolute quantitative RT-PCR analysis. Significance was assessed using one-way ANOVA. Mean levels ± SD from three independent experiments were plotted. The *p* values were considered significant at *p* < 0.05; **p* < 0.05, ***p* < 0.01, ****p* < 0.001.

**Figure S2**


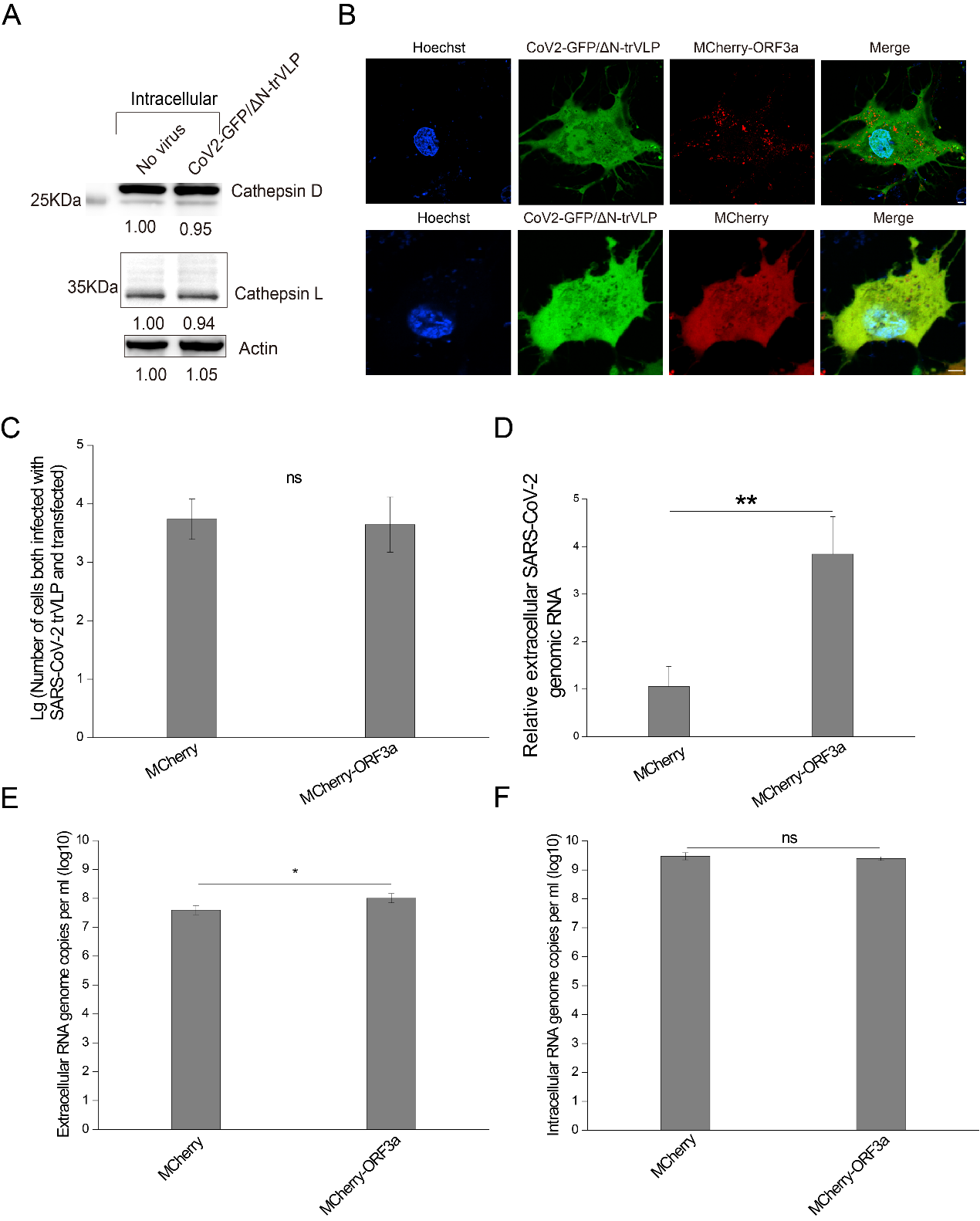


**Supplementary Figure S2.** **SARS-CoV-2 ORF3a protein promotes SARS-CoV-2 egress. Related to Figure 3.**

(A) Quantitative analysis of cathepsin D and cathepsin L in uninfected and SARS-CoV-2-trVLP-infected Caco-2-N cells. Representative immunoblot analyses of intracellular lysosomal cathepsin D and cathepsin L of uninfected and SARS-CoV-2-trVLP-infected cells are shown. The levels of cathepsin D and cathepsin L in control cells were both set to 1.0. (B) Representative confocal microscopy images show cells that are simultaneously infected with SARS-CoV-2 trVLP and transfected with mCherry or mCherry-ORF3a. Scale bars, 5 µm. (C) Quantitative analysis of the number of cells that are simultaneously infected SARS-CoV-2 trVLP and transfected with mCherry or mCherry-ORF3a. (D) The effect of SARS-CoV-2 ORF3a protein on the egress of SARS-CoV-2 trVLP. Extracellular SARS-CoV-2 genomic RNA levels in SARS-CoV-2-trVLP-infected mCherry-expressing and mCherry-ORF3a-expressing cells were quantified by relative quantitative RT-PCR analysis. SARS-CoV-2 genomic RNA levels were plotted as the relative Nsp12 copy number, normalized to the housekeeping gene GAPDH Mean levels ± SD from three independent experiments were plotted. (E, F) The effect of SARS-CoV-2 ORF3a protein on the genomic replication and egress of SARS-CoV-2 trVLP using absolute quantitative RT-PCR analysis. Extracellular (E) and intracellular (F) SARS-CoV-2 genomic RNA levels in SARS-CoV-2-trVLP-infected mCherry-expressing and mCherry-ORF3a-expressing cells were quantified. A serial dilution of the quantified plasmid (pcDNA3.1-Nsp12) was used as standard template for generating the standard curve. Mean levels ± SD from three independent experiments were plotted. Significance was assessed using unpaired two-tailed t-test. The *p* values were considered significant at *p* < 0.05; **p* < 0.05, ***p* < 0.01, ****p* < 0.001.

**Figure S3**


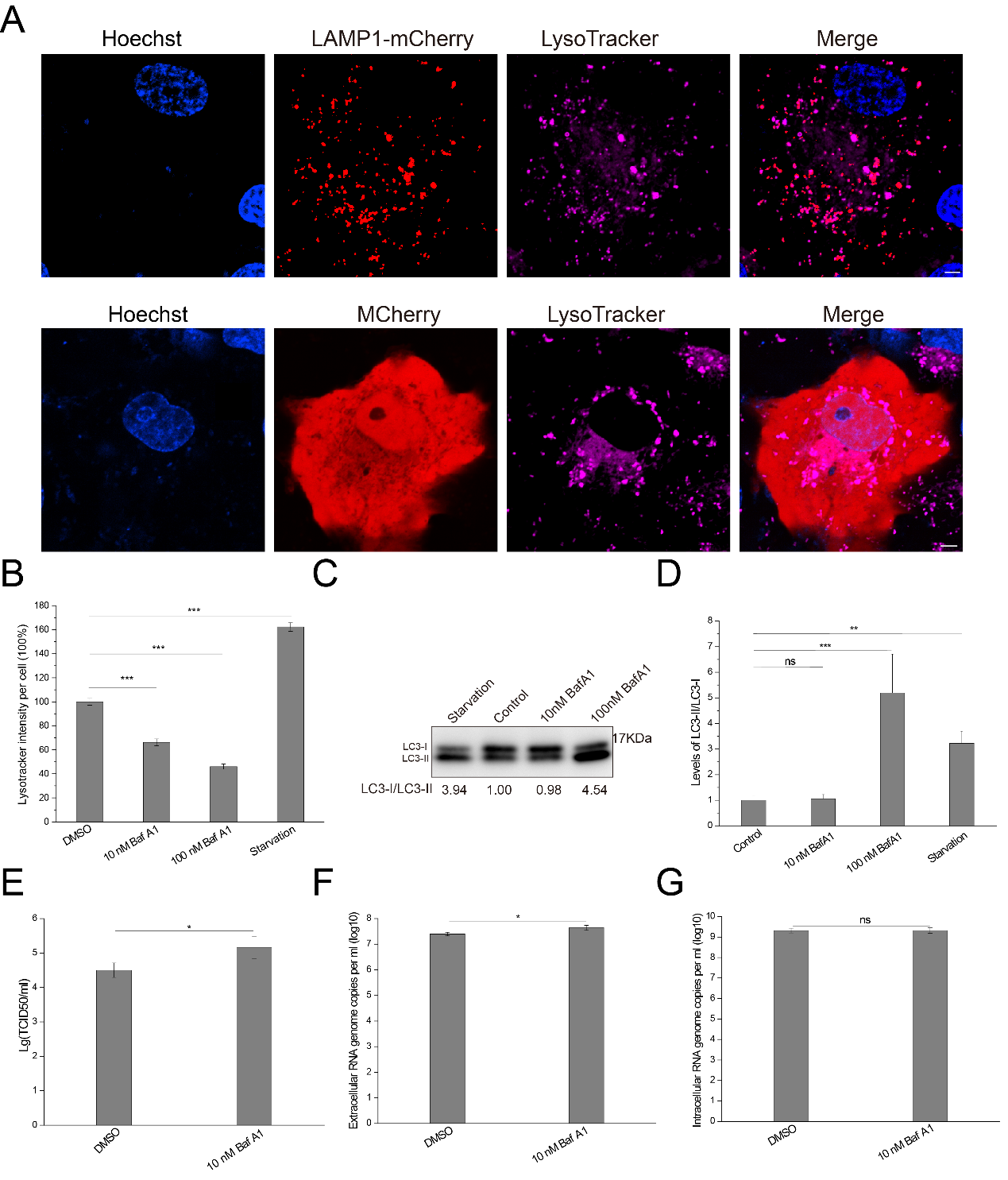


**Supplementary Figure S3. Lysosome deacidification facilitates SARS-CoV-2 egress. Related to Figure 4.**

(A) Representative images of cells expressing lysosome marker LAMP1 and stained with Lysotracker. To assess the efficiency of Lysotracker Deep Red in labeling lysosomes, cells expressing LAMP1-mCherry or mCherry (control) were stained with Lysotracker Deep Red. Scale bars, 5 µm. (B) Evaluation of the effect of bafilomycin A1-treatment on lysosomal pH. The fluorescence intensity of LysoTracker in DMSO treated cells, serving as the control group, was normalized to 100%. Mean levels ± SD from three independent experiments. (C) Evaluation of the effect of bafilomycin A1-treatment on autophagic flux by Western blot analysis of the LC3-II/LC3-I ratio. The level of LC3-II/LC3-I ratio in DMSO treated cells, serving as the control group, was set to 1.0. (D) Quantitative analysis of the effect of bafilomycin A1-treatment on autophagic flux. Mean levels ± SD from three independent experiments. (E) Effects of bafilomycin A1 treatment on the egress of SARS-CoV-2 trVLP by TCID50 assay. The levels of extracellular infectious SARS-CoV-2 trVLP in DMSO- and bafilomycin A1-treated SARS-CoV-2-trVLP-infected cells at 8–14 hpi were quantified by TCID50 assay. Mean levels ± SD from three independent experiments. (F, G) The effects of further lysosomal deacidification on the genomic replication and egress of SARS-CoV-2 trVLP using absolute quantitative RT-PCR analysis. Extracellular (F) and intracellular (G) SARS-CoV-2 genomic RNA levels in DMSO- and bafilomycin A1-treated (10 nM) SARS-CoV-2-trVLP-infected cells at 8–14 hpi were quantified. A serial dilution of the quantified plasmid (pcDNA3.1-Nsp12) was used as standard template for generating the standard curve. Significance was assessed using unpaired one-way ANOVA (B and D) and two-tailed t-test (E-G). Mean levels ± SD from three independent experiments were plotted. The *p* values were considered significant at *p* < 0.05; **p* < 0.05, ***p* < 0.01, ****p* < 0.001.

**Figure S4**


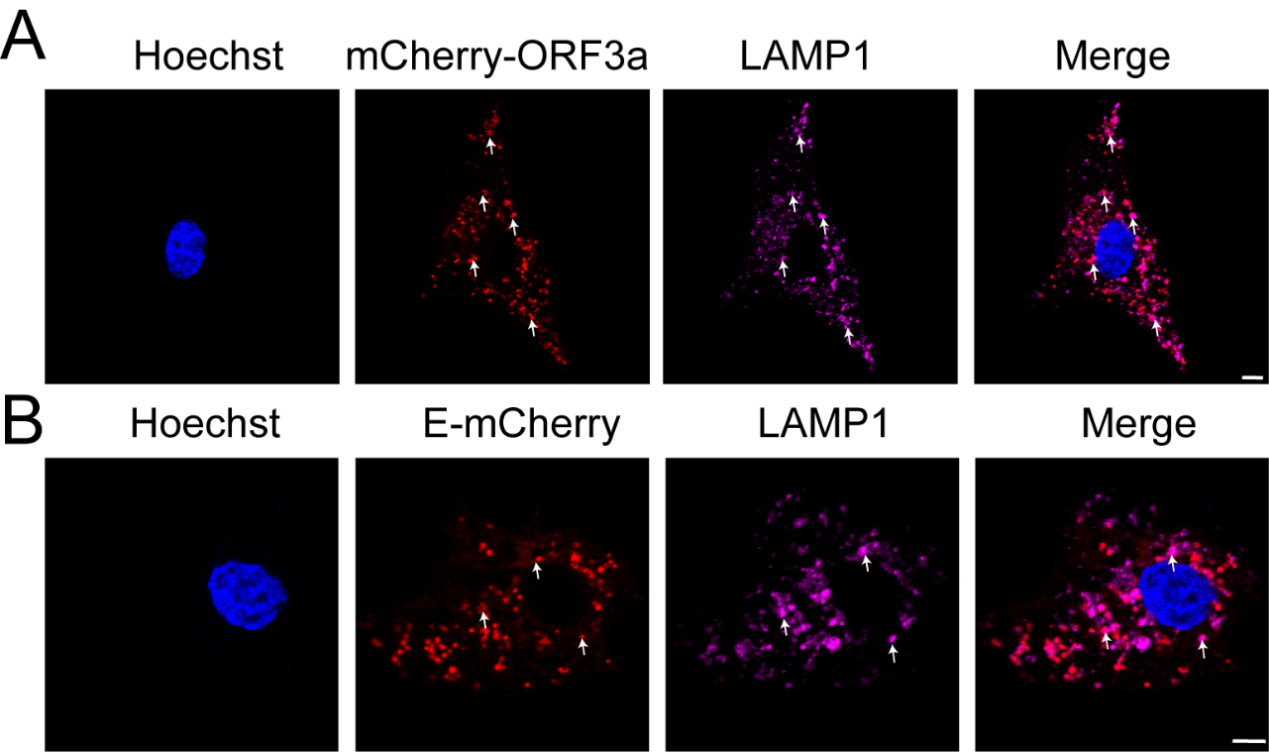


**Supplementary Figure S4. The ORF3a and E proteins of SARS-CoV-2 are colocalized with lysosomes. Related to Figure 5.**

(A) SARS-CoV-2 ORF3a protein is colocalized with lysosomes. Representation confocal microscopy images of mCherry-ORF3a colocalized with lysosome marker LAMP1immunostained with LAMP1 antibody. The arrows indicate the colocalization of mCherry-ORF3a with the lysosome marker LAMP1. Scale bar, 5 μm. (B) SARS-CoV-2 E protein is colocalized with lysosomes. Representation confocal microscopy images of E-mCherry colocalized with lysosome marker LAMP1 immunostained with LAMP1 antibody. The arrows indicate the colocalization of mCherry-ORF3a with the lysosome marker LAMP1. Scale bar, 5 μm.

**Figure S5**


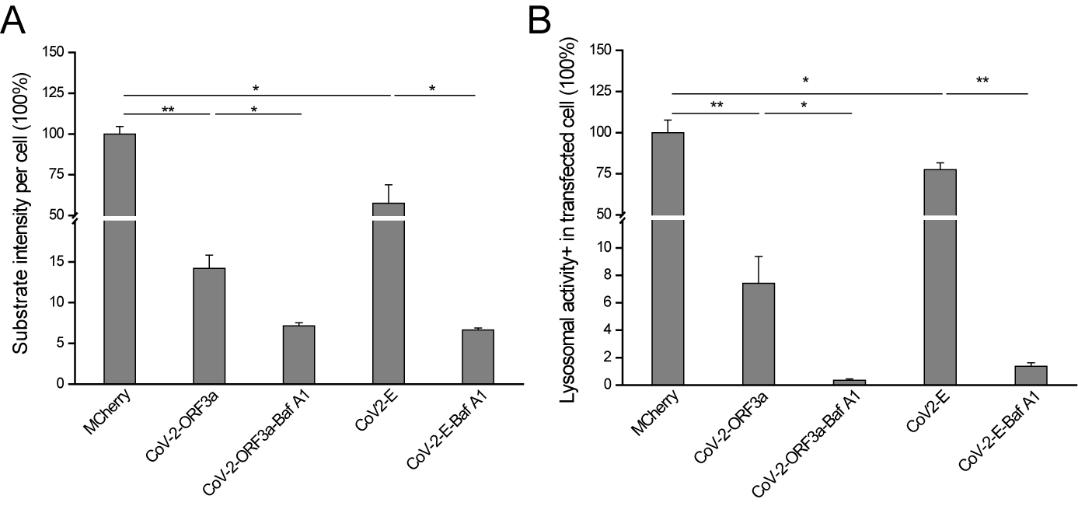


**Supplementary Figure S5. The ORF3a and E proteins of SARS-CoV-2 reduce the activities of lysosomal enzymes. Related to Figure 6.**

(A) Quantitative analysis of the fluorescence intensity of the substrates in cells expressing SARS-CoV-2 ORF3a or E protein, as quantified by flow cytometry. The fluorescence intensity of substrates in MCherry-transfected Vero E6 cells, serving as the control group, was normalized to 100%. The impact of ORF3a and E protein expression on the fluorescence intensity of substrates in these cells was then evaluated in comparison to this control. (B) Quantitative analysis of the proportion of lysosomal activity-positive cells among the cells expressing SARS-CoV-2 ORF3a or E protein under flow cytometry. The fluorescence intensity of substrates in mCherry-transfected Vero E6 cells, serving as the control group, was normalized to 100%. The impact of ORF3a and E protein expression on the fluorescence intensity of substrates in these cells was then evaluated in comparison to this control. Mean levels ± SD from experiments performed in triplicate were plotted. Significance was assessed using one-way ANOVA. The *p* values were considered significant at *p* < 0.05; **p* < 0.05, ***p* < 0.01, ****p* < 0.001

**Supplementary Table S1. The materials and resource presented in this study.**

| REAGENT or RESOURCE | SOURCE | IDENTIFIER |
| --- | --- | --- |
| Antibodies | | |
| Anti-LAMP1 antibody  (1:500, WB) | abcam | Cat#ab24170; RRID:AB_775978 |
| beta Actin Loading Control Monoclonal Antibody (BA3R), (1:3000, WB) | Thermo Scientific | Cat#MA5-15739;  RRID:AB_10979409 |
| Anti-Cathepsin D antibody [CTD-19](1:1000, WB) | abcam | Cat#ab6313;  RRID:AB_305416 |
| Rabbit Anti-Mouse IgG H&L (HRP) (1:3000, WB) | abcam | Cat#ab6728;  RRID:AB_955440 |
| Goat Anti-Rabbit IgG H&L (HRP) (1:3000, WB) | abcam | Cat#ab6721;  RRID:AB_955447 |
| Anti-Cathepsin L/V/K/H antibody [EPR8011] | abcam | Cat#ab133641;  RRID:AB_2630341 |
| Anti-LAMP1 antibody [H4A3] (1:20, IF) | abcam | Cat#ab25630;  RRID:AB_470708 |
| Goat Anti-Mouse IgG H&L (Alexa Fluor® 647),  (1:500, IF) | abcam | Cat#ab150115;  RRID:AB_2687948 |
| Anti-LAMP1 (extracellular epitope) (1:20, IF) | R&D Systems | Cat#AF4800;  RRID:AB_1026176 |
| NorthernLights™ Anti-sheep IgG-NL557(1:200, IF) | R&D Systems | Cat#NL010;  RRID:AB_884220 |
| LC3B antibody | Beyotime | Cat#AL221 |
| Anti-SARS-CoV-2 (COVID-19) Envelope antibody | Sigma-Aldrich | SAB3501136 |
| Anti-SARS CoV-2 ORF3a Protein antibody | abcam | ab280953;  RRID:AB_3675414 |
| Anti-SARS-CoV-2 (COVID-19) NSP8 antibody | Sigma-Aldrich | SAB3501131;  RRID:AB_2938787 |
| Bacterial and virus strains | | |
| SARS-CoV-2 GFP/ΔN trVLPs | Laboratory of Ding, Tsinghua University, Beijing |  |
| Chemicals, peptides, and recombinant proteins | | |
| Lysosome-Specific  Self-Quenched Substrate | Abcam | Cat.#Ab234622 |
| LysoTracker Deep Red | Thermo Scientific | Cat#L12492 |
| BAPTA-AM | Sigma | Cat#A4926 |
| CID1067700 | Sigma | Cat#314042-01-8 |
| Paraformaldehyde | Sigma | Cat#P6148 |
| BFA | Sigma | Cat.# 20350-15-6 |
| Dyngo-4a | Sigma | Cat.# S7163 |
| Bafilomycin A1 | MedChemExpress | HY-100558 |
| Critical commercial assays | | |
| BCA Protein Assay Kit | Thermo Fisher | Cat#23225 |
| Trans-Blot Turbo 5X Buffer | Bio Rad | Cat#10026938 |
| DMEM | Thermo Fisher | Cat#21063029 |
| Cathepsin L Activity  Assay kit (Fluorometric) | Abcam | Cat#ab65306 |
| Cathepsin S Activity  Assay Kit (Fluorometric) | Abcam | Cat#ab65307 |
| RNaseZap™ RNase Decontamination Solution | Thermo Fisher | Cat#AM9782 |
| Opti-MEM | GIBCO | Cat#31985070 |
| DMEM | GIBCO | Cat#C11995500BT |
| PBS | GIBCO | Cat#C10010500BT |
| FBS | Gibco | Cat# 10099-141C |
| Halt™ Protease and Phosphatase Inhibitor Cocktail | Thermo Fisher | Cat#78442 |
| 4–12% gradient tris-glycine gels | ACE | Cat#F11412Gel |
| HiPure Total RNA Kit | Magen | Cat#R4111-03 |
| HiPure viral RNA Kit | Magen | Cat#R4171-03 |
| HiScript® II U+ One Step qRT-PCR Probe Kit | Vazyme | Cat#Q222-01 |
| Experimental models: Cell lines | | |
| Vero E6 | Laboratory of Zhang, Shenzhen Institute of Advanced Technology, Chinese Academy of Sciences, Shenzhen | RRID:CVCL_0574 |
| Caco-2 | Laboratory of Zhang, Shenzhen Institute of Advanced Technology, Chinese Academy of Sciences, Shenzhen | RRID:CVCL_0025 |
| HEK293T | Laboratory of Zhang, Shenzhen Institute of Advanced Technology, Chinese Academy of Sciences, Shenzhen | RRID:CVCL_0063 |
| Caco-2-N | Laboratory of Ding, Tsinghua University, Beijing |  |
| Oligonucleotides | | |
| Forward primer for nsp12: GTGARATGGTCATGTGTGGCGG | This paper | N/A |
| Reverse primer for nsp12: CARATGTTAAASACACTATTAGCATA | This paper | N/A |
| TaqMan probe for nsp12: CAGGTGGAACCTCATCAGGAGATGC | This paper | N/A |
| Forward primer for GAPDH: CAACGGATTTGGTCGTATTGG | This paper | N/A |
| Primer for GAPDH: GCAACAATATCCACTTTACCAGAGTTAA | This paper | N/A |
| TaqMan probe for GAPDH: CGCCTGGTCACCAGGGCTGC | This paper | N/A |
| Recombinant DNA | | |
| pcDNA3.1-nsp1-mCherry | This paper | N/A |
| pcDNA3.1-nsp2-mCherry | This paper | N/A |
| pcDNA3.1-nsp3-mCherry | This paper | N/A |
| pcDNA3.1-nsp4-mCherry | This paper | N/A |
| pcDNA3.1-nsp5-mCherry | This paper | N/A |
| pcDNA3.1-nsp6-mCherry | This paper | N/A |
| pcDNA3.1-nsp7-mCherry | This paper | N/A |
| pcDNA3.1-nsp8-mCherry | This paper | N/A |
| pcDNA3.1-nsp9-mCherry | This paper | N/A |
| pcDNA3.1-nsp10-mCherry | This paper | N/A |
| pcDNA3.1-nsp11-mCherry | This paper | N/A |
| pcDNA3.1-nsp12-mCherry | This paper | N/A |
| pcDNA3.1-nsp13-mCherry | This paper | N/A |
| pcDNA3.1-nsp14-mCherry | This paper | N/A |
| pcDNA3.1-nsp15-mCherry | This paper | N/A |
| pcDNA3.1-nsp16-mCherry | This paper | N/A |
| pcDNA3.1-S-mCherry | This paper | N/A |
| pcDNA3.1-mCherry-ORF3a | This paper | N/A |
| pcDNA3.1-E-mCherry | This paper | N/A |
| pcDNA3.1-M-mCherry | This paper | N/A |
| pcDNA3.1-ORF6-mCherry | This paper | N/A |
| pcDNA3.1-ORF7a-mCherry | This paper | N/A |
| pcDNA3.1-ORF7b-mCherry | This paper | N/A |
| pcDNA3.1-ORF8-mCherry | This paper | N/A |
| pcDNA3.1-ORF9-mCherry | This paper | N/A |
| pcDNA3.1-ORF10-mCherry | This paper | N/A |
| pcDNA3.1-mCherry-ORF3a(∆[11–14]-L15V) | This paper | N/A |
| pcDNA3.1-mCherry-ORF3a(G196V) | This paper | N/A |
| pcDNA3.1-mCherry-ORF3a(T223I) | This paper | N/A |
| pcDNA3.1-mCherry-ORF3a(G251V) | This paper | N/A |
| pcDNA3.1-mCherry-ORF3a(S253P) | This paper | N/A |
| pLenti-EF1a-SARS2 N-Flag-BSD | Laboratory of Ding, Tsinghua University, Beijing, China | 11 |
| psPAX2 | Laboratory of Zhang, Shenzhen Institute of Advanced Technology, Chinese Academy of Sciences, Shenzhen, China | RRID:Addgene_12260 |
| pMD2.G | Laboratory of Zhang, Shenzhen Institute of Advanced Technology, Chinese Academy of Sciences, Shenzhen, China | RRID:Addgene_12259 |
| pcDNA3.1-mCherry-LAMP1 | This paper | N/A |
| Software and algorithms | | |
| Fiji (ImageJ) version 1.8 | N/A | https://imagej.net/Fiji |
| GraphPad PRISM 8 | GraphPad Software, Inc | https://www.graphpad.com |
| SnapGene | Insightful Science | https://www.snapgene.com/ |
| qPCRsoft4.1 | Analytik Jena AG | http://www.analytik-jena.com.cn |
| Origin 2019 | Origin Lab | https://www.originlab.com/2019 |
